# The acute effect of fasted exercise on energy intake, energy expenditure, subjective hunger and gastrointestinal hormone release compared to fed exercise in healthy individuals: a systematic review and network meta-analysis

**DOI:** 10.1038/s41366-021-00993-1

**Published:** 2021-11-03

**Authors:** James Frampton, Robert M. Edinburgh, Henry B. Ogden, Javier T. Gonzalez, Edward S. Chambers

**Affiliations:** 1 Section for Nutrition Research, Department of Metabolism, Digestion and Reproduction, Faculty of Medicine, Imperial College London, London, UK; 2 Section of Endocrinology and Investigative Medicine, Department of Metabolism, Digestion and Reproduction, Faculty of Medicine, Imperial College London, London, UK; 3 Department for Health, University of Bath, Bath, UK; 4 Faculty of Sport, Health and Wellbeing, Plymouth Marjon University, Plymouth, UK; 5 Centre for Nutrition, Exercise and Metabolism, University of Bath, Bath, UK

**Keywords:** Obesity, Obesity, Obesity, Nutrition

## Abstract

**Objective:**

To determine the acute effect of fasted and fed exercise on energy intake, energy expenditure, subjective hunger and gastrointestinal hormone release.

**Methods:**

CENTRAL, Embase, MEDLINE, PsycInfo, PubMed, Scopus and Web of Science databases were searched to identify randomised, crossover studies in healthy individuals that compared the following interventions: (i) fasted exercise with a standardised post-exercise meal [FastEx + Meal], (ii) fasted exercise without a standardised post-exercise meal [FastEx + NoMeal], (iii) fed exercise with a standardised post-exercise meal [FedEx + Meal], (iv) fed exercise without a standardised post-exercise meal [FedEx + NoMeal]. Studies must have measured ad libitum meal energy intake, within-lab energy intake, 24-h energy intake, energy expenditure, subjective hunger, acyl-ghrelin, peptide YY, and/or glucagon-like peptide 1. Random-effect network meta-analyses were performed for outcomes containing ≥5 studies.

**Results:**

17 published articles (23 studies) were identified. Ad libitum meal energy intake was significantly lower during FedEx + Meal compared to FedEx + NoMeal (MD: −489 kJ; 95% CI, −898 to −80 kJ; *P* = 0.019). Within-lab energy intake was significantly lower during FastEx + NoMeal compared to FedEx + NoMeal (MD: −1326 kJ; 95% CI, −2102 to −550 kJ; *P* = 0.001). Similarly, 24-h energy intake following FastEx + NoMeal was significantly lower than FedEx + NoMeal (MD: −2095 kJ; 95% CI, −3910 kJ to −280 kJ; *P* = 0.024). Energy expenditure was however significantly lower during FastEx + NoMeal compared to FedEx+NoMeal (MD: −0.67 kJ/min; 95% CI, −1.10 to −0.23 kJ/min; *P* = 0.003). Subjective hunger was significantly higher during FastEx + Meal (MD: 13 mm; 95% CI, 5–21 mm; *P* = 0.001) and FastEx + NoMeal (MD: 23 mm; 95% CI, 16–30 mm; *P* < 0.001) compared to FedEx + NoMeal.

**Conclusion:**

FastEx + NoMeal appears to be the most effective strategy to produce a short-term decrease in energy intake, but also results in increased hunger and lowered energy expenditure. Concerns regarding experimental design however lower the confidence in these findings, necessitating future research to rectify these issues when investigating exercise meal timing and energy balance.

**PROSPERO registration number:**

CRD42020208041.

**Key points:**

- Fed exercise with a standardised post-exercise meal resulted in the lowest energy intake at the ad libitum meal served following exercise completion.
- Fasted exercise without a standardised post-exercise meal resulted in the lowest within-lab and 24-h energy intake, but also produced the lowest energy expenditure and highest hunger.
- Methodological issues lower the confidence in these findings and necessitate future work to address identified problems

## Introduction

Obesity poses substantial health and economic burdens to society [1–3]. At a fundamental level, obesity is characterised by a chronic imbalance between energy intake and energy expenditure, in which intake exceeds expenditure [4]. Increasing energy expenditure in the form of exercise is consequently recommended to prevent and/or reverse bodyweight gain [5]. However, long-term exercise training typically produces modest reductions in bodyweight in individuals with obesity, especially when compared to dietary energy restriction [6]. Indeed, achieved bodyweight loss is often considerably lower than predicted [7], with large inter-individual variation in bodyweight loss response also being reported [8]. While increasing physical activity energy expenditure may induce compensatory reductions in other components of total energy expenditure [9], the discrepancy between achieved and predicted bodyweight loss has been primarily attributed to compensatory responses in energy intake [10].

Over the past decade, research has begun to focus on the influence of meal timing and provision on the physiological responses to exercise, in particular the effects of exercise performed in the postabsorptive state (fasted exercise) compared to exercise performed in the postprandial state (fed exercise). Thus far, systematic reviews have concentrated on differences in energy metabolism between fasted and fed exercise, concluding that fasted exercise produces significantly higher rates of fat oxidation [11, 12]. This finding may have important implications for appetite regulation, as altered energy expenditure and substrate utilisation are putative regulators of compensatory eating following exercise [13]. Alternatively, meal provision and acute exercise both influence the release of gastrointestinal hormones related to satiety [14, 15], which could interact to influence subjective appetite and feeding behaviour in an additive or synergistic manner.

We, therefore, conducted a systematic review and network meta-analysis to quantify the acute effect of fasted exercise relative to fed exercise on energy intake, energy expenditure, subjective hunger and gastrointestinal hormone release in healthy individuals. As fasted exercise is compared to fed exercise in multiple variants (fasted or fed exercise, with or without a standardised post-exercise meal) in the literature, a network meta-analysis was necessary in order to make comparisons between all four possible interventions. The results from this review and analysis will improve the understanding of the relationship between meal timing and provision relative to exercise on short-term energy balance, and thus better inform exercise guidelines for the regulation of bodyweight. In addition, this review will also help to identify limitations of the current body of work investigating diet-exercise interactions and consequently provide direction for future research.

## Methods

### Registration

This Review and Network meta-analysis was registered at PROSPERO (registration number: CRD42020208041). This manuscript was prepared in line with the guidance outlined in the PRISMA statement for systematic reviews and network meta-analyses [16].

### Eligibility criteria

#### Inclusion criteria

Studies were included if they were randomised and employed a crossover study design comparing the effects of a single bout of exercise performed in the postabsorptive state (fasted exercise; ≥6 h following meal ingestion; with and without a standardised post-exercise meal) with a single exercise bout performed in the postprandial state (fed exercise; <6 h following meal ingestion; with and without a standardised post-exercise meal) on energy intake and/or energy expenditure and/or subjective hunger and/or gastrointestinal hormone (acyl-ghrelin, glucagon-like peptide 1, peptide YY) release. Studies directly comparing fasted exercise with and without a standardised post-exercise meal and fed exercise with and without a standardised post-exercise meal were also included.

**Fig. 1:**
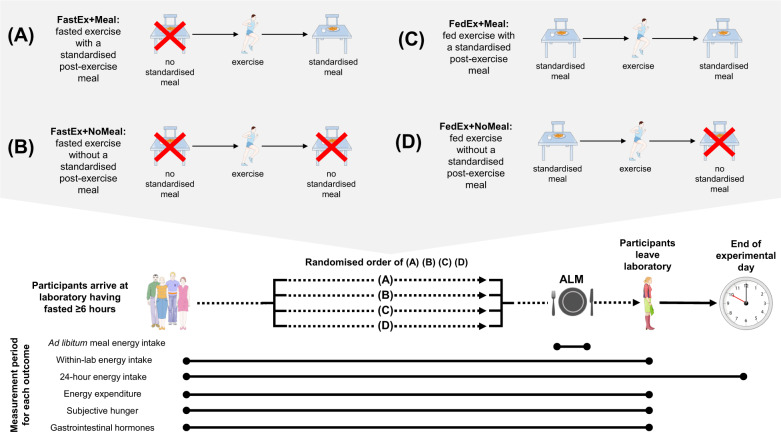
Overview of study design variants for fasted and fed exercise interventions currently used in research. Participants typically arrived at the laboratory following an overnight fast and undergo one of four interventions (**A**: FastEx + Meal, **B**: FastEx + NoMeal, **C**: FedEx + Meal, **D**: FedEx + NoMeal), after which an ad libitum meal can be provided. Energy expenditure, subjective hunger and gastrointestinal hormone concentrations via serial blood sampling are often measured at regular interval throughout the laboratory visit. After the meal participants are free to leave the laboratory, and may be asked to keep a record of what they eat for the remainder of the day to calculate 24-h energy intake. Participants would then return to the lab on a separate day and following an adequate washout period to undergo one of the three remaining interventions. Note: participants would normally only complete two exercise interventions: one fasted (FastEx + Meal or FastEx+NoMeal) and one fed (FedEx + Meal or FedEx+NoMeal). ALM, ad libitum meal.

This inclusion criteria created four groups for comparison (Fig. 1):

1. Fasted exercise with a standardised post-exercise meal (FastEx + Meal): Exercise performed ≥6 h following last meal, with a standardised meal ingested within ≤1 hour of exercise completion and prior to the consumption of an ad libitum meal (if provided).
2. Fasted exercise without a standardised post-exercise meal (FastEx + NoMeal): Exercise performed ≥6 h following last meal, with no standardised meal ingested within ≤1 h of exercise completion and prior to the consumption of an ad libitum meal (if provided).
3. Fed exercise with a standardised post-exercise meal (FedEx + Meal): Exercise performed <6 h following a standardised meal, with a standardised meal also ingested within ≤1 h of exercise completion and prior to the consumption of an ad libitum meal (if provided).
4. Fed exercise without a standardised post-exercise meal (FedEx + NoMeal): Exercise performed <6 h following a standardised meal, with no standardised meal ingested within ≤1 h of exercise completion and prior to the consumption of an ad libitum meal (if provided).

Within each study, exercise sessions must have been matched for intensity, duration and modality and performed at approximately the same time of day (±3 h). Fed exercise must have been performed ≥10 min after meal ingestion. All standardised meals must have contained a minimum of 418.4 kJ (100 kcal). Participants could be of any age, body mass index (BMI) value and fitness level.

#### Exclusion criteria

Studies were excluded if they were not written in the English language, not published in a peer-reviewed journal or included a performance-based (e.g. time trial) or exhaustive (e.g. participants instructed to exercise until voluntary exhaustion) exercise session. Studies in which participants consumed food or energy-containing beverages during the exercise session were also excluded. Participants that were smokers, pregnant, taking any medication that might interfere with appetite, or had a history of chronic disease, including diabetes, were also excluded. Studies were also excluded if participants ingested caffeine, alcohol, or a pharmaceutical agent at any point during the experimental session. This was necessary to prevent these factors from confounding the effect of the interventions on included outcomes. For the purpose of this review, participants that met the inclusion/exclusion criteria were defined as ‘healthy’.

If a study stated that a measurement of a relevant outcome had been conducted but the results were not included in the published manuscript or if the results of a relevant outcome were presented inadequately, corresponding authors were contacted for data provision. If authors did not respond or could not provide the requested data, and no other relevant outcomes were reported in the manuscript, the study was excluded.

#### Definition of outcomes

Ad libitum meal energy intake: total energy intake at the first ad libitum meal presented to participants following exercise completion (measured in kJ or kcal).

Within-lab energy intake: total energy intake consumed in the laboratory environment (including energy intake consumed from both standardised and ad libitum meals; measured in kJ or kcal).

24-h energy intake: total energy intake on the day of the experimental session (including any energy consumed during the experimental session; measured in kJ or kcal).

Energy expenditure: total energy expenditure measured for the full experimental session in the laboratory environment (measured in kJ or kcal) or energy expenditure assessed at three or more timepoints in the laboratory environment (measured in kJ/min or kcal/min).

Subjective hunger: assessed by a 100 mm visual analogue scale (VAS) at three or more timepoints using a question referencing hunger (e.g. “how hungry do you feel?”, or similar) in the laboratory environment (measured in mm or cm).

Gastrointestinal hormones: acyl-ghrelin, glucagon-like peptide1 and/or peptide YY assessed in serum or plasma at three or more timepoints in the laboratory environment (measured in SI or conventional units).

### Search strategy

The Cochrane Central Register of Controlled Trials (CENTRAL), Embase, MEDLINE, PsycInfo, PubMed, Scopus and Web of Science databases were searched from inception to July 2020 without limits using the search terms ‘carbohydrate’, ‘breakfast’, ‘postprandial’, ‘fed’, ‘feeding’,’fast’, ‘fasted’, ‘fasting’, ‘skipping’, ‘omission’, ‘low glycogen’, ‘glycogen depletion’, ‘carbohydrate loading’, ‘glucose deprived’, ‘low glucose’, ‘exercise’, ‘exercising’, ‘physical activity’, ‘walk’, ‘walking’, ‘run’, ‘running’, ‘cycle’, ‘cycling’, ‘crossover’, ‘crossover’, ‘counterbalanced’, ‘acute’, ‘randomised’, ‘randomised’, ‘appetite’, ‘energy’, ‘glucagon-like peptide 1’, ‘glp-1’, ‘glp1’, ‘peptide tyrosine tyrosine’, ‘peptide YY’, ‘PYY’, and ‘ghrelin’. The full search strategy is provided in Supplementary Appendix S1.

### Screening process

Database results were imported into Covidence systematic review software (Veritas Health Innovation, Australia). Title and abstract screening were first conducted independently by three authors (J.F, R.M.E, H.B.O), in which studies were classified as either ‘yes’, ‘no’ or ‘maybe’. Each study required two ‘yes’ votes in order to progress to the full-text screening phase. Full-text screening was conducted by the same three authors (J.F, R.M.E, H.B.O) with studies being classified as ‘yes’ or ‘no’. Again, studies that received two ‘yes’ votes proceeded to the data extraction phase. Any disagreements in study classification during the screening process were discussed and resolved before proceeding to the next screening phase.

### Data extraction

Data from included studies were extracted by a single author (J.F) into an electronic spreadsheet (Excel 2016, Microsoft Corporation, USA) and checked for accuracy by a second author (E.S.C) prior to analysis. The following data were obtained: authors, year of publication, sample size, participant characteristics (age, BMI, maximal aerobic capacity), intervention characteristics (energy content of meal, carbohydrate content of meal, fat content of meal, protein content of meal), and exercise bout characteristics (mode, intensity, duration).

Ad libitum meal energy intake and/or within-lab energy intake and/or 24-h energy intake with corresponding standard deviations for fasted and fed exercise arms were also imputed. Time-averaged area under the curve (AUC) values and standard deviations were calculated for energy expenditure, subjective hunger, acyl-ghrelin, glucagon-like peptide 1 and peptide YY for both fasted and fed exercise arms to allow for comparisons between studies that measured these outcomes over different time periods. Time-averaged AUC values and corresponding standard deviations for these outcomes were then entered into the electronic spreadsheet. Corresponding authors of studies reporting at least one of these outcomes were first contacted asking for the raw data. If the raw data were provided this was used to calculate time-averaged AUC. If authors could not provide the raw data or did not respond to the data request, WebPlotDigitizer Version 4.2 (Ankit Rohatgi, USA) was used to extract data from figures within the manuscript.

Energy intake (kJ), energy expenditure (kJ/min), subjective hunger (mm) acyl-ghrelin (pmol/L), glucagon-like peptide 1 (pmol/L) and peptide YY (pmol/L) values were converted into a common unit of measurement (see parentheses), with SI units being used where appropriate. Mean differences (MD) between fasted (FastEx + Meal or FastEx+NoMeal) and fed exercise (FedEx + Meal or FedEx + NoMeal) arms for all outcomes were calculated by subtracting fed exercise values from fasted exercise values. Therefore, a positive value would indicate an increase with fasted exercise, and a negative value would indicate a decrease with fasted exercise. For comparisons between fasted exercise interventions (FastEx + Meal or FastEx + NoMeal), FastEx + Meal values were subtracted from FastEx + NoMeal values. For comparisons between fed exercise interventions (FedEx + Meal or FedEx + NoMeal), FedEx + Meal values were subtracted from FedEx + NoMeal values. A correlation coefficient of 0.5 was assumed in order to calculate standard error when within-participant correlation coefficients were unavailable [17].

### Risk of bias evaluation

Risk of bias was evaluated using the Cochrane Risk of Bias tool for randomised controlled trials with additional considerations for crossover trials. Additional considerations comprised carryover effects, period effects, and concerns that trials may report only analyses based on the first period. Risk of bias was assessed under the following domains: bias arising from the randomisation process; bias due to deviations from intended intervention; bias due to missing outcome data; bias in the measurement of the outcome; and bias in selection of the reported result. Evaluations were carried out independently for each outcome. Studies measuring multiple outcomes, therefore, had multiple classifications (one for each outcome measured). No studies were excluded based on the risk of bias assessment.

### Meta-analysis procedures

Data were imported into R (version 4.0.2) for analysis. Data included: sample size, mean difference and corresponding standard error of the mean difference.

Random-effects network meta-analyses were performed using the statistical package ‘netmeta’ in R within a frequentist framework [18]. Network meta-analyses are able to compare three or more interventions using both direct and indirect evidence [19]. Direct evidence is obtained from direct comparisons between two interventions. For example, direct evidence for the difference between treatment A and treatment B would be taken from studies directly comparing treatment A versus treatment B. This is identical to the approach used in traditional pairwise meta-analyses. In contrast, indirect evidence is obtained from indirect comparisons based on a shared comparator. For example, indirect evidence for the difference between treatment A and treatment B could be obtained from studies comparing treatment A versus treatment C, and treatment B versus treatment C (the shared comparator being treatment C). For a more detailed explanation of network meta-analyses, we refer the reader to chapter 11 of the Cochrane Handbook for Systematic Reviews of Interventions [20].

There are several advantages of using network meta-analyses over pairwise meta-analyses. Firstly, effect estimates calculated using direct and indirect evidence (referred to as combined estimates) possess higher precision than effect estimates derived from traditional pairwise meta-analyses [21]. Secondly, network meta-analyses can use indirect evidence to calculate effect estimates for comparisons that were not performed in any available study [21]. Effect estimates based only on indirect evidence are referred to as indirect estimates. Lastly, network-meta-analyses can correctly incorporate studies that contain multiple arms, avoiding the problem of double-counting of participants [22].

Pooled mean differences with 95% confidence intervals were calculated for each outcome with interventions being ranked based on their P-score [23]. Network geometry was explored using a network graph, in which node size is proportional to the number of participants that undertook that intervention, and line thickness is proportional to the number of studies directly comparing the connected interventions. Local incoherence was assessed using the Separating Indirect from Direct Evidence (SIDE; also referred to as node splitting) approach [24]. Global incoherence was assessed using the I^2^ statistic. The I^2^ statistic ranges from 0% to 100%, with values of 25% to 49%, 50% to 74% and ≥75% classified as low, moderate, and high, respectively [25]. Comparison-adjusted funnel plots were generated for each outcome to assess publication bias, with statistical assessment by Egger’s test also being conducted [26]. Results for all analyses are displayed as mean effects sizes with 95% confidence intervals (CI) and statistical significance was set as *P* < 0.05.

No statistical analyses were performed for outcomes that contained an insufficient number of studies (<5 studies).

### Confidence in evidence evaluation

Confidence in evidence was evaluated for all effect estimates using the Confidence In Network Meta‐Analysis (CINeMA) framework as described by Papakonstantinou et al. [27]. This method evaluates the confidence in evidence using the following domains: within‐study bias; reporting bias; indirectness; imprecision; heterogeneity; and incoherence. The estimated effect for each comparison within each outcome was then categorised as very low, low, moderate or high confidence.

## Results

**Fig. 2:**
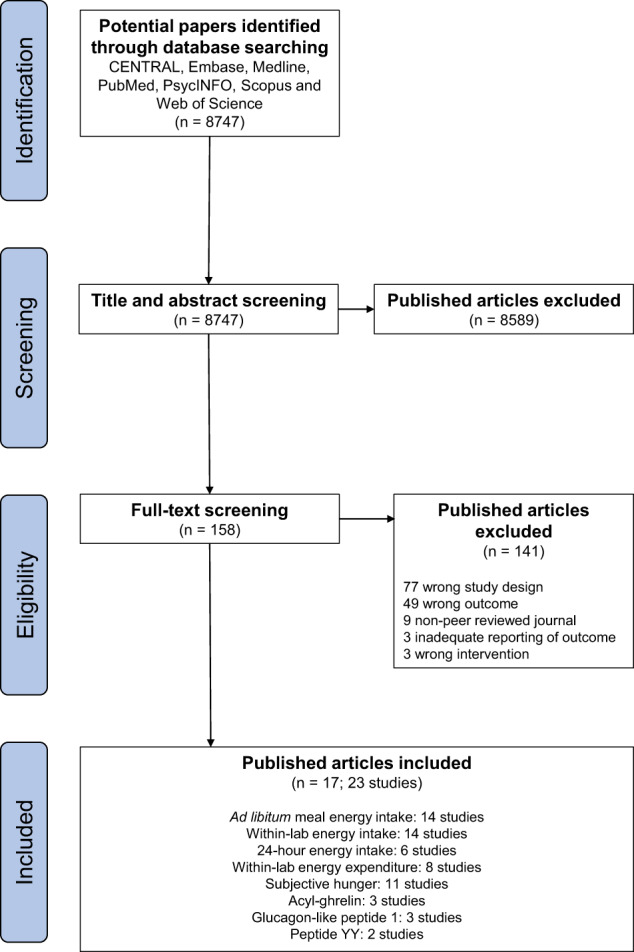
PRISMA flow diagram. Flow diagram depicting process of paper selection.

Database searches identified 8747 potentially eligible published articles. Following title and abstract screening, 158 published articles underwent full-text screening, resulting in 17 published articles being deemed eligible. Due to several published articles containing multiple studies, a total of 23 separate studies were included in the analysis. Each outcome consisted of the following number of studies and participants––ad libitum meal energy intake: 14 studies, 217 participants; within-lab energy intake: 14 studies, 217 participants; 24-h energy intake: six studies, 83 participants; energy expenditure: eight studies, 69 participants; subjective hunger: 11 studies, 145 participants; acyl-ghrelin: three studies, 36 participants; glucagon-like peptide 1: three studies, 37 participants; peptide YY: two studies, 24 participants. The screening process is summarised in Fig. 2.

Ad libitum meal energy intake, within-lab energy intake, 24-h energy intake, energy expenditure, and subjective hunger outcomes contained ≥5 studies and therefore a network meta-analysis was performed. Acyl-ghrelin, glucagon-like peptide 1 and peptide YY contained 3, 3, and 2 studies, respectively; therefore only descriptive data is provided.

### Participant and intervention characteristics

Participant characteristics, intervention characteristic and reported outcomes for included studies are summarised in Table 1.

**Table 1:** Participant and intervention characteristics of included studies.

Author	Participant characteristics	Comparison(s)	Standardised meal characteristics(pre-exercise)	Standardised meal characteristics (post-exercise)	Exercise characteristics	Meal-Exercise & Exercise-Meal interval (minutes)	Outcomes reported
Bachman et al. [51]	12 males	FastEx + NoMeal vs FedEx + NoMeal	2298 kJ	N/A	treadmill	120	ad libitum meal energy intake, within-lab energy intake, 24-h energy intake, subjective hunger
	Age: 20.8 ± 3.0		114 g CHO, 7 g FAT, 14 g PRO		60 mins	- - - - - - -	
	BMI: 22.7 ± 2.6				60% V̇O_2_ max	N/A	
	V̇O_2_ max: 59.1 ± 5.7						
Bennard & Doucet [52]^a^	8 males	FastEx + Meal vs FedEx + NoMeal	1674 kJ	1674 kJ	treadmill	45	energy expenditure
	Age: 24.5 ± 2.1		80 g CHO	80 g CHO	42 mins	- - - - - - -	
	BMI: 23.8 ± 1.9				50% V̇O_2_ max	20	
	V̇O_2_ max: 54.0 ± 5.4						
Bennard & Doucet [52]^b^	8 males	FastEx + Meal vs FedEx + NoMeal	1674 kJ	1674 kJ	treadmill	45	energy expenditure
	Age: 24.5 ± 2.1		80 g CHO	80 g CHO	42 mins	- - - - - - -	
	BMI: 23.8 ± 1.9				50% V̇O_2_ max	20	
	V̇O_2_ max: 54.0 ± 5.4						
Broad et al. [53]	10 males	FastEx + Meal vs FedEx + NoMeal	2440 kJ	2440 kJ	treadmill	60	24-h energy intake, energy expenditure
	Age: 21.3 ± 2.0		108 g CHO, 12 g FAT, 21 g PRO	108 g CHO, 12 g FAT, 21 g PRO	8 ×15 secs, 120 secs rest	- - - - - - -	
	BMI: 25.7 ± 2.9				All out	30	
	V̇O_2_ max: 48.3 ± 3.5						
Brown et al. [54]	13 females	FedEx + Meal vs FedEx + NoMeal	10% daily energy requirements	1360 kJ	cycle ergometer	180	ad libitum meal energy intake, within-lab energy intake, subjective hunger, glucagon-like peptide 1
	Age: 23.0 ± 4.0			78.6 g CHO	60 mins	- - - - - - -	
	BMI: 23.1 ± 2.9				65% V̇O_2_ max	0	
	V̇O_2_ max: 43.5 ± 11.6						
Cheng et al. [55]	12 males	FastEx + Meal vs FedEx + NoMeal	5675 kJ	5675 kJ	cycle ergometer	120	subjective hunger, peptide YY
	Age: 24.6 ± 4.8		88 g CHO, 105 g FAT, 14 g PRO	88 g CHO, 105 g FAT, 14 g PRO	50 mins	- - - - - - -	
	V̇O_2_ max: 59.2 ± 8.1				60% V̇O_2_ max	60	
Clayton et al. [56]	12 males	FedEx + Meal vs FedEx + NoMeal	1810 kJ	529 kJ	cycle ergometer	120	ad libitum meal energy intake, within-lab energy intake
	Age: 24.0 ± 2.0			31 g CHO	60 mins	- - - - - - -	
	BMI: 23.2 ± 1.4				30 mins at 65% V̇O_2_ max,	10	
	V̇O_2_ max: 52.0 ± 8.0				5 ×3 min at 85% V̇O_2_ max, 2 min rest		
Davis et al. [57]^a^	7 females	FastEx + NoMeal vs FedEx + NoMeal	3807 kJ	3807 kJ	treadmill	20	energy expenditure
	Age: 24.0 ± 2.4		132 g CHO, 22 g FAT, 45 g PRO	132 g CHO, 22 g FAT, 45 g PRO	20 mins	- - - - - - -	
	V̇O_2_ max: 49.9 ± 1.8				60% V̇O_2_ max	10	
Davis et al. [57]^b^	7 females	FastEx + Meal vs FedEx + NoMeal	3807 kJ	3807 kJ	treadmill	20	energy expenditure
	Age: 24.0 ± 2.4		132 g CHO, 22 g FAT, 45 g PRO	132 g CHO, 22 g FAT, 45 g PRO	20 mins	- - - - - - -	
	V̇O_2_ max: 49.9 ± 1.8				60% V̇O_2_ max	10	
Davis et al. [57]^c^	7 females	FastEx + NoMeal vs FastEx + Meal	3807 kJ	3807 kJ	treadmill	20	energy expenditure
	Age: 24.0 ± 2.4		132 g CHO, 22 g FAT, 45 g PRO	132 g CHO, 22 g FAT, 45 g PRO	20 mins	- - - - - - -	
	V̇O_2_ max: 49.9 ± 1.8				60% V̇O_2_ max	10	
Edinburgh et al. [32]	12 males	FastEx + Meal vs FedEx + Meal	1803 kJ	1255 kJ	cycle ergometer	120	ad libitum meal energy intake, within-lab energy intake, 24-h energy intake, energy expenditure
	Age: 23.0 ± 3		65 g CHO, 11 g FAT, 19 g PRO	75 g CHO	60 mins	- - - - - - -	
	BMI: 23.6 ± 2.0				50% PPO	0	
	V̇O_2_ max: 53.0 ± 10.0						
Farah & Gill [58]	10 males	FastEx + Meal vs FedEx + NoMeal	1927 kJ	1927 kJ	treadmill	30	ad libitum meal energy intake, within-lab energy intake, energy expenditure, subjective hunger
	Age: 28.1 ± 10.7		56 g CHO, 19 g FAT, 16 g PRO	56 g CHO, 19 g FAT, 16 g PRO	60 mins	- - - - - - -	
					50% V̇O_2_ max	30	
	BMI: 29.0 ± 2.8						
	V̇O_2_ max: 39.1 ± 5.4						
Gonzalez et al. [59]	12 males	FastEx + Meal vs FedEx + Meal	1857 kJ	1500 kJ	treadmill	120	ad libitum meal energy intake, within-lab energy intake, subjective hunger, glucagon-like peptide 1
	Age: 23.2 ± 4.3		67 g CHO, 11 g FAT, 19 g PRO	56 g CHO, 8 g FAT, 16 g PRO	59 mins	- - - - - - -	
	V̇O_2_ max: 53.1 ± 5.5				61% V̇O_2_ max	20	
Griffiths et al. [60]^a^	12 males	FastEx + NoMeal vs FedEx + NoMeal	2238 kJ	N/A	treadmill	60	ad libitum meal energy intake, within-lab energy intake, subjective hunger, acyl-ghrelin
	Age: 23.0 ± 3.0		78 g CHO, 14 g FAT, 24 g PRO		60 mins	- - - - - - -	
	V̇O_2_ max: 53.0 ± 8.6				40%, 50%, 60% V̇O_2_ max	N/A	
					(20 min each)		
Griffiths et al. [60]^b^	12 males	FastEx + NoMeal vs FedEx + NoMeal	2238 kJ	N/A	treadmill	60	ad libitum meal energy intake, within-lab energy intake, subjective hunger, acyl-ghrelin
	Age: 23.0 ± 3.0		78 g CHO, 14 g FAT, 24 g PRO		60 mins	- - - - - - -	
	V̇O_2_ max: 53.0 ± 8.6				40%, 50%, 60% V̇O_2_ max	N/A	
					(20 min each)		
Hunschede et al. [61]	30 males	FastEx + NoMeal vs FedEx + NoMeal	952 kJ	N/A	treadmill	10	ad libitum meal energy intake, within-lab energy intake
	Age: 17.2 ± 3.3		57 g CHO		3 ×20 mins, 5 mins rest	- - - - - - -	
	V̇O_2_ max: 43.5 ± 9.0				47% V̇O_2_ max	N/A	
McIver et al. [62]	12 males	FastEx + Meal vs FedEx + Meal	733 kJ	1013 kJ	treadmill	60	subjective hunger, acyl-ghrelin, glucagon-like peptide 1, peptide YY
	Age: 26.0 ± 5.0		30 g CHO, 3 g FAT, 7 g PRO	25 g CHO, 12 g FAT, 8 g PRO	45 mins	- - - - - - -	
	BMI: 27.0 ± 4.0				50% V̇O_2_ max	30	
	V̇O_2_ max: 39.0 ± 6.0						
McIver et al. [63]	12 males	FastEx + Meal vs FedEx + Meal	1438 kJ	1584 kJ	treadmill	60	24-h energy intake, subjective hunger
	Age: 25.0 ± 3.0		48 g CHO,10 g FAT, 11 g PRO	66 g CHO, 7 g FAT, 9 g PRO	45 mins	- - - - - - -	
	BMI: 26.0 ± 4.0				55% V̇O_2_ max	30	
	V̇O_2_ max: 39.0 ± 4.0						
Tamam et al. [64]^a^	18 males	FedEx + Meal vs FedEx + NoMeal	1272 kJ	745 kJ	treadmill	120	ad libitum meal energy intake, within-lab energy intake
	Age: 12.4 ± 1.3			45 g CHO	15 mins	- - - - - - -	
	V̇O_2_ max: 21.6 ± 3.0				VT	5	
Tamam et al. [64]^b^	17 males	FedEx + Meal vs FedEx + NoMeal	1272 kJ	854 kJ	treadmill	120	ad libitum meal energy intake, within-lab energy intake
	Age: 11.0 ± 1.2			51 g CHO	15 mins	- - - - - - -	
	V̇O_2_ max: 19.3 ± 4.1				VT	5	
Tamam et al. [64]^c^	19 males	FedEx + Meal vs FedEx + NoMeal	1272 kJ	782 kJ	treadmill	120	ad libitum meal energy intake, within-lab energy intake
	Age: 12.4 ± 1.7			47 g CHO	15 mins	- - - - - - -	
	V̇O_2_ max: 21.4 ± 2.6				25% above VT	5	
Thivel et al. [65]	6 males, 8 females	FedEx + Meal vs FedEx + NoMeal	2092 kJ	741 kJ	cycle ergometer	105	ad libitum meal energy intake, within-lab energy intake, subjective hunger
	Age: 12.8 ± 0.9				30 mins	- - - - - - -	
	BMI: 34.8 ± 5.7				65% V̇O_2_ max	0	
	V̇O_2_ max: 22.3 ± 4.2						
Veasey et al. [43]	24 females	FastEx + NoMeal vs FedEx + NoMeal	984 kJ	N/A	treadmill	45	ad libitum meal energy intake, within-lab energy intake, 24-h energy intake, subjective hunger
	Age: 20.9 ± 2.3		38 g CHO, 3.6 g FAT, 9 g PRO		30 mins	- - - - - - -	
	BMI: 21.9 ± 1.9				65% HRR	N/A	

^a.b.c^ denote sub-studies. Participant characteristic (units): years (age), BMI (kg/m^2^) and V̇O_2_ max (ml/min/kg).

*Meal-Exercise interval* the time between the consumption of a standardised meal and commencement of exercise (number above dotted line), *Exercise-Meal interval* the time between completion of exercise and consumption of a standardised meal (number below dotted line), *FastEx* *+* *Meal* fasted exercise with a standardised meal, *FastEx* *+* *NoMeal* fasted exercise without a standardised meal, *FedEx* *+* *Meal* fed exercise with a standardised meal, *FedtEx* *+* *NoMeal* fed exercise without a standardised meal, *CHO* carbohydrate, *HRR* heart rate reserve, *N/A* not applicable, *PPO* peak power output, *PRO* protein, *VT* ventilatory threshold.

Of the 23 eligible studies, only six included females (five exclusively, one also including males) with the remaining 17 studies including only males. All studies were conducted in young individuals with mean participant age ranging from 11 to 28 years old, of which five were conducted in children (<18 years old; four exclusively, one also including adults). Seven studies included lean participants (BMI < 25 kg/m^2^), five studies included overweight participants (BMI ≥ 25 kg/m^2^), and eleven studies did not report BMI values.

Six studies compared FastEx + NoMeal to FedEx + NoMeal, six studies compared FastEx + Meal to FedEx + NoMeal, four studies compared FastEx + Meal to FedEx + Meal, six studies compared FedEx + Meal to FedEx + NoMeal and one study compared FastEx + Meal, with FastEx + NoMeal and FedEx + NoMeal.

The standardised meal provided before exercise typically contained foods associated with breakfast, with porridge (*n* = 7) and cereal with milk (*n* = 8) being the most popular options. The energy content of this standardised meal ranged from 733 kJ to 5,675 kJ (175–1,356 kcal), was provided between 20 and 180 min before exercise, and was typically high in carbohydrates (>50% of total energy; *n* = 16).

The standardised meal provided after exercise was typically a high-carbohydrate beverage (>50% of total energy; *n* = 13). The energy content of this standardised meal ranged from 529 kJ to 5675 kJ (126 kcal to 1356 kcal) and was given from immediately post-exercise to 60 min post-exercise.

Most studies used a treadmill (*n* = 18) for the exercise intervention, with the remaining studies using a cycle ergometer (*n* = 5). Exercise was largely conducted at a fixed intensity (*n* = 19), lasting between 40 and 60 min (*n* = 13), and performed at a moderate intensity (40–60% V̇O_2_ max; *n* = 14).

### Risk of bias analysis

Risk of bias summary tables for each outcome is presented in Supplementary Appendix S2. For studies measuring ad libitum meal energy intake and within-lab energy intake, 50% of studies had a high risk of bias (Figure S1A). Similarly, 67% of studies measuring 24-h energy intake also had a high risk of bias (Figure S1B). Most studies measuring energy expenditure (75%) had an unclear risk of bias (Figure S1C), whereas the majority of studies measuring subjective hunger (64%) had a high risk of bias (Figure S1D). All studies measuring gastrointestinal hormones (acyl-ghrelin, glucagon-like peptide 1, peptide YY) had an unclear risk of bias (Figures S2,A–C).

### Ad libitum meal energy intake

There were five studies comparing FastEx + NoMeal to FedEx + NoMeal, one study comparing FastEx + Meal to FedEx + NoMeal, two studies comparing FastEx + Meal to FedEx + Meal, and six studies comparing FedEx + Meal to FedEx + NoMeal. There were no studies comparing FastEx + NoMeal to FedEx + Meal or FastEx + NoMeal to FastEx + Meal (Figure S3,A). Effect estimates and confidence intervals for each study are presented in Figure S4.

**Fig. 3:**
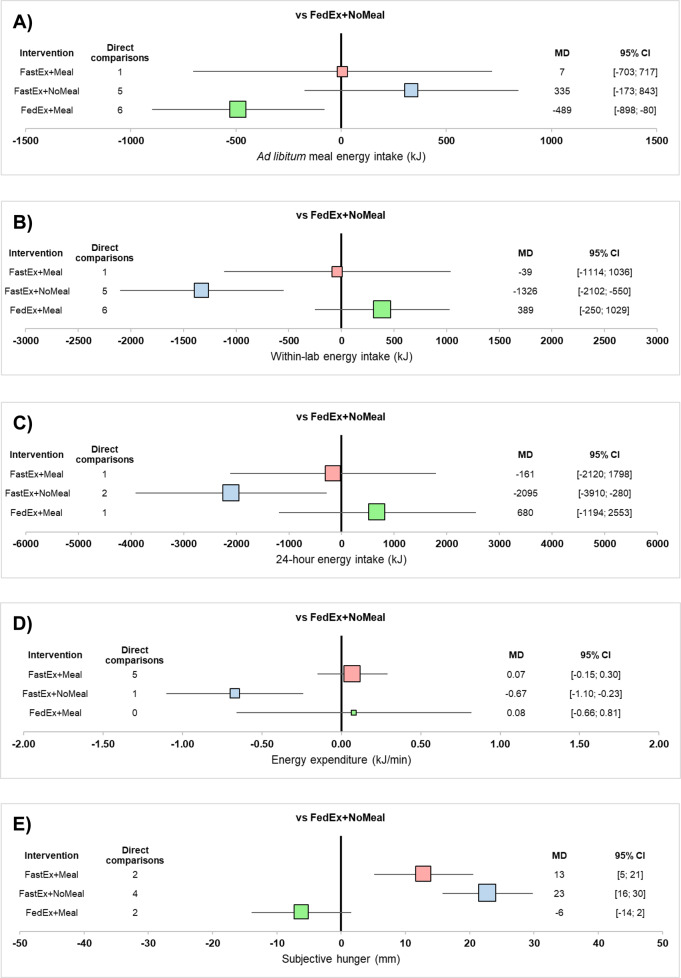
Summary of effects. Forest plots of effect estimates for (**A**) ad libitum meal energy intake, (**B**) within-lab energy intake, (**C**) 24-h energy intake, (**D**) energy expenditure, and (**E**) subjective hunger. Data are presented as mean differences (MD) ± 95% confidence intervals (CI). FastEx+Meal, fasted exercise with a standardised post-exercise meal; FastEx+NoMeal, fasted exercise without a standardised post-exercise meal; FedEx + Meal, fed exercise with a standardised post-exercise meal; FedEx+NoMeal, fed exercise without a standardised post-exercise meal.

Results from the network meta-analysis revealed that FedEx + Meal was ranked as the most effective intervention at reducing ad libitum meal energy intake (P-score = 0.970; Supplementary Appendix S3) and was significantly lower than FedEx + NoMeal (MD: −489 kJ; 95% CI, −898 to −80 kJ; P = 0.019; Fig. 3A). No significant differences in ad libitum meal energy intake were detected between FastEx + Meal and FedEx + NoMeal (MD: 7 kJ; 95% CI, −703 to 717 kJ; *P* = 0.985) or FastEx + NoMeal and FedEx + NoMeal (MD: 335 kJ; 95% CI, −173 to 843 kJ; *P* = 0.196). There was also no significant difference in ad libitum meal energy intake between FastEx + Meal and FedEx + Meal (MD: 496 kJ; 95% CI, −173 to 1165 kJ; *P* = 0.146; Supplementary Appendix S4).

Comparisons based on only indirect evidence showed that ad libitum energy intake was significantly higher during FastEx + NoMeal compared to FedEx + Meal (MD: 824 kJ; 95% CI, 172 to 1477 kJ; *P* = 0.013). Indirect evidence also showed no significant difference in ad libitum energy intake between FastEx + Meal and FastEx + NoMeal (MD: −328 kJ; 95% CI, −1201 to 545 kJ; *P* = 0.461; Supplementary Appendix S4).

Results from the SIDE approach showed no significant differences between direct and indirect evidence, suggesting no substantial level of local incoherence (Supplementary Appendix S5). There was however a moderate level of global incoherence (*I*^*2*^ = 56.5%). The comparison-adjusted funnel plot did not suggest evidence of publication bias (Figure S5,A), which was supported by results from Egger’s test (*P* = 0.383).

### Within-lab energy intake

There were five studies comparing FastEx + NoMeal to FedEx + NoMeal, one study comparing FastEx + Meal to FedEx + NoMeal, two studies comparing FastEx + Meal to FedEx + Meal, and six studies comparing FedEx + Meal to FedEx + NoMeal. There were no studies comparing FastEx + NoMeal to FedEx + Meal or FastEx + NoMeal to FastEx + Meal (Figure S3,B). Effect estimates and confidence intervals for each study are presented in Figure S6.

Results from the network meta-analysis found that FastEx + NoMeal was the most effective intervention at reducing within-lab energy intake (P-score = 0.990; Supplementary Appendix S3) and was significantly lower than FedEx + NoMeal (MD: −1326 kJ; 95% CI, −2102 to −550 kJ; *P* = 0.001; Fig. 3B). There were no significant differences in within-lab energy intake between FastEx + Meal and FedEx + NoMeal (MD: −39 kJ; 95% CI, −1114 to 1036 kJ; *P* = 0.944) or FedEx + Meal and FedEx + NoMeal (MD: 389 kJ; 95% CI, −250 to 1029 kJ; *P* = 0.232). Similarly, within-lab energy intake did not significantly differ between FastEx + Meal and FedEx + Meal (MD: −428 kJ; 95% CI, −1441 to 584 kJ; *P* = 0.407; Supplementary Appendix S4).

Results based on only indirect evidence revealed that within-lab energy intake was significantly lower during FastEx + NoMeal compared to FedEx + Meal (MD: −1715 kJ; 95% CI, −2721 to −710 kJ; *P* = 0.001), but not significant difference between FastEx + Meal and FastEx + NoMeal (MD: 1287 kJ; 95% CI, −39 to 2613 kJ; *P* = 0.057; Supplementary Appendix S4).

Results from the SIDE approach showed no significant differences between direct and indirect evidence, suggesting no substantial level of local incoherence (Supplementary Appendix S5). Nevertheless, there was a high level of global incoherence (*I*^*2*^ = 81.9%). The comparison-adjusted funnel plot (Fig. S5,B) and the results from Egger’s test (*P* = 0.517) did not suggest evidence of publication bias.

### 24-h energy intake

Two studies compared FastEx + NoMeal to FedEx + NoMeal, one study compared FastEx + Meal to FedEx + NoMeal, two studies compared FastEx + Meal to FedEx + Meal, and one study compared FedEx + Meal and FedEx + NoMeal. There were no studies comparing FastEx + NoMeal to FedEx + Meal or FastEx + NoMeal to FastEx + Meal (Fig. S3,C). Effect estimates and confidence intervals for each study are presented in Figure S7.

Results from the network meta-analysis revealed that FastEx+NoMeal was the most effective intervention at reducing 24-h energy intake (P-score = 0.964; Supplementary Appendix S3) and was significantly lower than FedEx + NoMeal (MD: −2095 kJ; 95% CI, −3910 to −280; *P* = 0.024; Fig. 3 C). However, no significant differences in 24-h energy intake between FastEx + Meal and FedEx + NoMeal (MD: −161 kJ; 95% CI, −2120 to 1798 kJ; *P* = 0.872), FedEx + Meal and FedEx + NoMeal (MD: 680 kJ; 95% CI, −1194 to 2553 kJ; *P* = 0.477), or FastEx + Meal and FedEx + Meal (MD: −841 kJ; 95% CI, −2361 to 679 kJ; *P* = 0.278; Supplementary Appendix S4) were detected.

Comparisons based on only indirect evidence found that 24-h energy intake was significantly lower during FastEx + NoMeal compared to FedEx + Meal (MD: −2775 kJ; 95% CI, −5383 to −166 kJ; *P* = 0.037). However, indirect evidence revealed no significant difference in 24-h energy intake between FastEx + Meal and FastEx + NoMeal (MD: 1934 kJ; −736 kJ to 4605 kJ; *P* = 0.156; Supplementary Appendix S4).

Results from the SIDE approach showed no significant differences between direct and indirect evidence, suggesting no substantial level of local incoherence (Supplementary Appendix S5). There was however a moderate level of global incoherence (I^2^ = 63.5%). The comparison-adjusted funnel plot did not suggest evidence of publication bias (Figure S5,C). Egger’s test was not performed because of the small number of studies reporting 24-h energy intake (*n* = 6).

### Energy expenditure

One study compared FastEx + NoMeal to FedEx + NoMeal, six studies compared FastEx + Meal to FedEx + NoMeal, one study compared FastEx + Meal to FedEx + Meal, and one study compared FastEx + NoMeal to FastEx + Meal. There were no studies comparing FastEx + NoMeal to FedEx + Meal or FedEx + Meal to FedEx + NoMeal (Fig. S3,D). Effect estimates and confidence intervals for each study are presented in Figure S8.

The results of the network meta-analysis revealed that FastEx + Meal is the most effective intervention at increasing energy expenditure (P-Score = 0.744; Supplementary Appendix S3) but was not significantly different from FedEx + NoMeal (MD: 0.07 kJ/min; 95% CI, −0.15 to 0.30 kJ/min; *P* = 0.529; Fig. 3D) or FedEx + Meal (MD: 0.00 kJ/min; 95% CI, −0.70 to 0.69 kJ/min; *P* = 0.992). Energy expenditure during FastEx + NoMeal was however significantly lower than during both FedEx + NoMeal (MD: −0.67 kJ/min; 95% CI, −1.10 to −0.23 kJ/min; *P* = 0.003) and FastEx + Meal (MD: −0.74 kJ/min; 95% CI, −1.18 to −0.31 kJ/min; *P* < 0.001; Supplementary Appendix S4).

Comparisons based on only indirect evidence showed that energy expenditure was not significantly different between FastEx + NoMeal and FedEx + Meal (MD: −0.75 kJ/min; 95% CI, −1.57 to 0.08 kJ/min; *P* = 0.075) or FedEx + Meal and FedEx + NoMeal (MD: 0.08 kJ/min; 95% CI, −0.66 to 0.81 kJ/min; *P* = 0.837; Supplementary Appendix S4).

Results from the SIDE approach showed no significant differences between direct and indirect evidence, suggesting no substantial level of local incoherence (Supplementary Appendix S5). There was however a high degree of global incoherence (*I*^*2*^ = 81.3%). There was no evidence of publication bias following a visual inspection of the comparison-adjusted funnel plot (Fig. S5,D). Egger’s test was not performed due to the small number of studies assessing energy expenditure (*n* = 8).

### Subjective hunger

Four studies compared FastEx + NoMeal to FedEx + NoMeal, two studies compared FastEx + Meal to FedEx + NoMeal, three studies compared FastEx + Meal to FedEx + Meal, and two studies compared FedEx + Meal to FedEx + NoMeal. There were no studies comparing FastEx + NoMeal to FedEx + Meal or FastEx + NoMeal to FastEx + Meal (Figure S3,E). Effect estimates and confidence intervals for each study are presented in Figure S9.

Results from the network meta-analyses found that FedEx + Meal was the most effective intervention at reducing subjective hunger (P-score = 0.980; Supplementary Appendix S3) but was not significantly different from FedEx + NoMeal (MD: −6 mm; 95% CI, −14 to 2 mm; *P* = 0.119; Fig. 3E). Compared to FedEx + NoMeal, subjective hunger was significantly higher during both FastEx + Meal (MD: 13 mm; 95% CI, 5 to 21 mm; *P* = 0.001) and FastEx + NoMeal (MD: 23 mm; 95% CI, 16 to 30 mm; *P* < 0.001). Likewise, subjective hunger was significantly higher during FastEx + Meal compared to FedEx + Meal (MD: 19 mm; 95% CI, 12 to 26 mm; *P* < 0.001; Supplementary Appendix S4).

Results based on only indirect evidence revealed that subjective hunger was significantly higher during FastEx + NoMeal compared to FedEx + Meal (MD: 29 mm; 95% CI, 18 to 39 mm; *P* < 0.001). However, indirect evidence found no significant difference between FastEx + Meal and FastEx + NoMeal (MD: −10 mm; −20 to 0 mm; *P* = 0.062; Supplementary Appendix S4)

Results from the SIDE approach showed no significant differences between direct and indirect evidence, suggesting no substantial level of local incoherence (Supplementary Appendix S5). Global incoherence was high (*I*^*2*^ = 76.5%) and visual inspection of the comparison-adjusted funnel plot did not indicate publication bias (Fig. S5,D). Egger’s test also suggested that publication bias was not present (*P* = 0.644).

### Gastrointestinal hormones

All three studies measuring acyl-ghrelin concentrations compared FastEx + NoMeal to FedEx + NoMeal. For glucagon-like peptide 1, two studies compared FastEx + Meal to FedEx + Meal and one study compared FedEx + Meal to FedEx + NoMeal. For peptide YY, one study compared FastEx + Meal to FedEx + NoMeal, and one other study compared FastEx + Meal to FedEx + Meal. Network meta-analysis were therefore not performed for these outcomes and only individual study effects are presented (Fig. S10-S12).

### Confidence in evidence

Confidence in evidence for all effect estimates is displayed in Table 2. Full details of the evaluation are provided in Supplementary Appendix S6.

**Table 2:** Summary of findings.

Ad libitum meal energy intake				
Total studies: 14	Relative effect (95% CI)	Confidence in evidence	Ranking	Interpretation of findings
Total participants: 217				
Fasted exercise with a standardised post-exercise meal (1 study; 10 participants)	7 kJ	⊕⊕⊝⊝		Unlikely inferior or superior
	(−703 to 717 kJ)	Low	3	
	Combined estimate	Due to within-study bias and imprecision^a^		
Fasted exercise without a standardised post-exercise meal (5 studies; 88 participants)	338 kJ	⊕⊕⊝⊝		Probably inferior
	(−174 to 850 kJ)	Low	4	
	Combined estimate	Due to within-study bias, imprecision and heterogeneity^b^		
Fed exercise with a standardised post-exercise meal (6 studies; 93 participants)	−489 kJ	⊕⊕⊝⊝		Probably superior
	(-898 to -79 kJ)	Low	1	
	Combined estimate	Due to within-study bias and heterogeneity^c^		
Fed exercise without a standardised post-exercise meal	Reference comparator	Reference comparator	2	Reference comparator
Within-lab energy intake				
Total studies: 14	Relative effect (95% CI)	Confidence in evidence	Ranking	Interpretation of findings
Total participants: 217				
Fasted exercise with a standardised post-exercise meal (1 study; 10 participants)	−39 kJ	⊕⊕⊝⊝		Unlikely inferior or superior
	(−1113 to 1035 kJ)	Low	3	
	Combined estimate	Due to within-study bias and imprecision^a^		
Fasted exercise without a standardised post-exercise meal (5 studies; 88 participants)	−1316 kJ	⊕⊕⊝⊝		Probably Superior
	(−2096 to −536 kJ)	Low	1	
	Combined estimate	Due to within-study bias and heterogeneity^d^		
Fed exercise with a standardised post-exercise meal (6 studies; 93 participants)	389 kJ	⊕⊕⊕⊝		Probably inferior
	(−249 to 1028 kJ)	Moderate	4	
	Combined estimate	Due to within-study bias, imprecision and heterogeneity^e^		
Fed exercise without a standardised post-exercise meal	Reference comparator	Reference comparator	2	Reference comparator
24-h energy intake				
Total studies: 6	Relative effect (95% CI)	Confidence in evidence	Ranking	Interpretation of findings
Total participants: 83				
Fasted exercise with a standardised post-exercise meal (1 study; 10 participants)	−161 kJ	⊕⊕⊝⊝		Unlikely inferior or superior
	(−2120 to 1798 kJ)	Low	2	
	Combined estimate	Due to within-study bias and imprecision^f^		
Fasted exercise without a standardised post-exercise meal (2 studies; 36 participants)	−2095 kJ	⊕⊕⊝⊝		Probably superior
	(−3910 to −280 kJ)	Low	1	
	Combined estimate	Due to within-study bias and heterogeneity^d^		
Fed exercise with a standardised post-exercise meal (1 studies; 13 participants)	680 kJ	⊕⊕⊝⊝		Unlikely inferior or superior
	(−1194 to 2553 kJ)	Low	4	
	Combined estimate	Due to within-study bias and imprecision^g^		
Fed exercise without a standardised post-exercise meal	Reference comparator	Reference comparator	3	Reference comparator
Energy expenditure				
Total studies: 6	Relative effect (95% CI)	Confidence in evidence	Ranking	Interpretation of findings
Total participants: 69				
Fasted exercise with a standardised post-exercise meal (5 studies; 43 participants)	0.07 kJ/min	⊕⊕⊕⊝		Unlikely inferior or superior
	(−0.15 to 0.30 kJ/min)	Moderate	1	
	Combined estimate	Due to within-study bias^h^		
Fasted exercise without a standardised post-exercise meal (1 study; 7 participants)	−0.67 kJ/min	⊕⊕⊕⊝		Probably inferior
	(−1.10 to −0.23 kJ/min)	Moderate	4	
	Combined estimate	Due to within-study bias^h^		
Fed exercise with a standardised post-exercise meal (0 studies; 0 participants)	0.08 kJ/min	⊕⊕⊕⊕	2	Unlikely inferior or superior
	(−0.66 to 0.81 kJ/min)	High		
	Indirect estimate			
Fed exercise without a standardised post-exercise meal	Reference comparator	Reference comparator	3	Reference comparator
Subjective hunger				
Total studies: 11	Relative effect (95% CI)	Confidence in evidence	Ranking	Interpretation of findings
Total participants: 145				
Fasted exercise with a standardised post-exercise meal (2 studies; 22 participants)	13 mm	⊕⊕⊕⊝		Probably inferior
	(5 to 21 mm)	Moderate	3	
	Combined estimate	Due to within-study bias and heterogenity^i^		
Fasted exercise without a standardised post-exercise meal (4 studies; 58 participants)	23 mm	⊕⊕⊝⊝		Probably inferior
	(16 to 30 mm)	Low	4	
	Combined estimate	Due to within-study bias^j^		
Fed exercise with a standardised post-exercise meal (2 studies; 27 participants)	−6 mm	⊕⊕⊝⊝		Probably superior
	(−14 to 2 mm)	Low	1	
	Combined estimate	Due to within-study bias, imprecision and heterogeneity^b^		
Fed exercise without a standardised post-exercise meal	Reference comparator	Reference comparator	2	Reference comparator

Estimates of effects, 95% confidence intervals, and certainty of the evidence for fasted exercise in healthy individuals

Patient or population: healthy individuals

Interventions: fasted exercise with a standardised post-exercise meal, fasted exercise without a standardised post-exercise meal, fed exercise with a standardised meal

Comparator (reference): fed exercise without a standardised post-exercise meal

Setting: laboratory environment

Summary of findings table definitions

*Estimates are expressed as mean differences. CI: confidence interval.

**Rankings are based on P-scores derived from the network meta-analyses.

***Interpretation of findings is in reference to fed exercise without a standardised post-exercise meal.

Confidence in evidence levels

High: we are very confident that the true effect lies close to that of the estimate of the effect.

Moderate: we are moderately confident in the effect estimate: the true effect is likely to be close to the estimate of the effect, but there is a possibility that it is substantially different.

Low: our confidence in the effect estimate is limited: the true effect may be substantially different from the estimate of the effect.

Very low: we have very little confidence in the effect estimate: the true effect is likely to be substantially different from the estimate of effect.

Explanatory footnotes

^a^Some concerns regarding within-study bias (unclear risk of bias in measurement of the outcome) and major concerns regarding imprecision (95% CI extends into clinically important effects in both directions).

^b^Major concerns regarding within-study bias (high risk of bias in measurement of the outcome), some concerns regarding imprecision (95% CI extends from clinically important effect to no effect), and some concerns regarding heterogeneity (prediction interval extends into clinically important or unimportant effects).

^c^Some concerns regarding within-study bias (unclear risk of bias arising from the randomisation process) and major concerns regarding heterogeneity (prediction interval extends into clinically important effects in both directions).

^d^Major concerns regarding within-study bias (high risk of bias in measurement of the outcome) and major concerns regarding heterogeneity (prediction interval extends into clinically important effects in both directions).

^e^Some concerns regarding within-study bias (unclear risk of bias arising from the randomisation process), some concerns regarding imprecision (95% CI extends from clinically important effect to no effect), and some concerns regarding heterogeneity (prediction interval extends into clinically important or unimportant effects).

^f^Some concerns regarding within-study bias (unclear risk of bias arising from the randomisation process and in measurement of the outcome) and major concerns regarding imprecision (95% CI extends into clinically important effects in both directions).

^g^Some concerns regarding within-study bias (unclear risk of bias arising from the randomisation process) and major concerns regarding imprecision (95% CI extends into clinically important effects in both directions).

^h^Some concerns regarding within-study bias (unclear risk of bias arising from the randomisation process).

^i^Some concerns regarding within-study bias (unclear risk of bias arising from the randomisation process and in measurement of the outcome) and heterogeneity (prediction interval extends into clinically important or unimportant effects).

^j^Major concerns regarding within-study bias (measurement of outcome).

## Discussion

The aim of this systematic review and network meta-analysis was to compare the effect of fasted and fed exercise with and without a standardised post-exercise meal on multiple components of energy balance. FedEx + Meal resulted in a significantly lower ad libitum meal energy intake than both FedEx + NoMeal and FastEx + NoMeal. However, when assessing within-lab energy intake, FastEx + NoMeal produced a significantly lower energy intake compared to both fed exercise interventions. This finding persisted outside of the laboratory environment, with 24-h energy intake being significantly lower during FastEx + NoMeal relative to both fed exercise interventions. Despite FastEx + NoMeal producing the greatest reduction in acute energy intake, energy expenditure was significantly lower during this intervention compared to both FedEx + NoMeal and FastEx + Meal. Subjective hunger was also significantly higher for both fasted exercise interventions relative to both fed exercise interventions.

It is not surprising that FedEx + Meal resulted in the lowest ad libitum meal energy intake, as this is the intervention that consumed the greatest amount of energy prior to the ad libitum meal (i.e. a standardised meal before and after exercise). Similarly, the finding that FastEx + NoMeal produced the greatest reduction in within-lab and 24-h energy intake was expected as this intervention provided no standardised meals during the experimental session. This is consistent with prior findings that meal omission is not fully compensated for at later ad libitum meals throughout the day [28, 29]. Reducing the number of hours available for eating during the day, a strategy that is akin to time-restricted feeding and inherent to the FastEx + NoMeal intervention, decreases spontaneous energy intake [30] and therefore may also contribute to the reduction in 24-h energy intake.

The initial aim of this review was to investigate possible differences in acyl-ghrelin, glucagon-like peptide 1, and peptide YY concentrations between fasted and fed because of their well-established roles in appetite regulation [31]. However, due to the paucity of studies measuring these hormones, their influence in mediating differences in energy intake between exercise interventions remains unknown. Consequently, future work should look to include these measures when exploring mechanistic differences between fasted and fed exercise.

It is also possible that the decrease in energy intake with FastEx + NoMeal is related to the elevated rates of fat oxidation produced by fasted exercise [11, 12], which can persist for up to 48-h post-exercise, and possibly decrease liver glycogen turnover [13]. Decreased liver glycogen turnover has been proposed as a mitigator of compensatory eating following exercise and thus may explain the lower energy intake following FastEx + NoMeal, potentially via increased secretion of fibroblast growth factor 21 [32, 33].

Energy expenditure was significantly lower with FastEx + NoMeal compared to both FedEx + NoMeal and FastEx + Meal. The reduced energy expenditure during FastEx + NoMeal is likely due to a decrease in diet-induced thermogenesis (as no standardised meals were provided), which is estimated to account for 5–15% of total energy expenditure [34]. Lastly, the greater sensations of subjective hunger during fasted exercise relative to fed exercise can likely be attributed to the differences in standardised meal energy intake between these interventions, as subjective appetite measures are highly responsive to meal ingestion [35, 36].

As the small decrease in energy expenditure during the FastEx + NoMeal experimental session is unlikely to offset the subsequent reduction in 24-h energy intake, these results suggest that FastEx + NoMeal is the most favourable intervention for inducing a short-term negative energy balance. However, there are several caveats that must be considered when interpreting and applying these findings to relevant populations.

Firstly, while the reduction in energy expenditure during FastEx + NoMeal may not considerably alter overall energy balance, it is possible that fasted exercise may result in reductions in energy expenditure outside of the laboratory environment where spontaneous movement is unrestricted. For example, breakfast omission is associated with a decrease in physical activity energy expenditure throughout the day relative to breakfast consumption, although this effect is predominantly during the period of morning fasting [37]. To our knowledge, there is no study that has investigated the effect of FastEx + NoMeal on subsequent spontaneous physical actively for the remainder of the day relative to fed exercise. Future work should therefore look to explore any potential impact of fasted exercise on energy expenditure in a free-living environment.

Secondly, when comparing FastEx + NoMeal to fed exercise interventions (FedEx + NoMeal or FedEx + Meal), it is impossible to determine whether fasted exercise per se influences an outcome, or if it is just the result of standardised meal omission (i.e. fasted and fed exercise interventions not being energy matched). Comparisons between FastEx + Meal and FedEx + NoMeal is thus the only true evaluation of fasted exercise alone (although the timing of the standardised meal is another factor to consider with this comparison). The present review found that subjective hunger was significantly lower during FedEx + NoMeal compared to FastEx + Meal, but this did not translate into any difference in the multiple facets of energy balance. Nonetheless, elevated sensations of hunger have been shown to influence food choices [38], and therefore the increase in hunger as a result of performing fasted exercise may cause individuals to make poor food choices e.g. selecting high-energy foods. This issue is less problematic with research undertaken in a controlled laboratory environment whereby participants have limited or no choice over which foods are consumed for most of the day. However, when performed in a free-living situation where individuals have full autonomy with respect to food choice, this may result in food choices that ultimately negate any energy deficit created by the exercise session. Whilst methodology is difficult, future research could look to investigate the influence of exercise performed in the fed versus fasted state on decisions regarding food selection.

Thirdly, all exercise sessions performed in included studies were supervised and therefore adherence to protocols was ensured. Likewise, training studies comparing fasted to fed exercise exclusively use supervised exercise sessions [39–42], and thus the impact of fasted relative to fed exercise on adherence to a training programme is largely unknown. Nevertheless, studies assessing ratings of perceived exertion and exercise enjoyment report no differences between fasted and fed exercise sessions [43–46], suggesting that training programme adherence would likely be similar irrespective of the metabolic state it was performed.

Finally, changes in energy balance over a 24-h period may however not necessarily translate to a chronic negative energy balance and weight loss. It is possible that the increase in subjective hunger apparently inherent to fasted exercise may result in a compensatory increase in energy intake beyond 24-h. As this period is little studied, the presence or absence of any compensatory response outside the initial 24 h is unknown. Most chronic studies (≥4 weeks) comparing fasted and fed exercise prescribe a diet in which energy intake is fixed [39–42], preventing any investigation of dietary energy compensation with this intervention. Furthermore, assessment of energy intake outside of the laboratory environment is commonly made using instruments that typically rely on self-reporting. These instruments are prone to misreporting [47, 48], undermining the confidence of any assessment of the impact of exercise interventions on free-living energy intake.

Limitations of the present review include the homogeneous nature of the included studies with respect to participant and exercise characteristics. Limited or no data were available regarding the effect of fasted and fed exercise on energy balance in females, the elderly, or in individuals with obesity, or using resistance exercise or high-intensity interval training. Similarly, very few studies measured acyl-ghrelin, glucagon-like peptide 1 or peptide YY concentrations, preventing any meta-analysis from being performed for these mechanistic outcomes. Most studies were also conducted in the morning, and therefore any interaction between time of day and intervention efficacy is unknown. This may be particularly relevant to fasted exercise, where improvements in metabolic health have been attributed to changes in lipid metabolism [46]; a parameter shown to be influenced by the time of day at which exercise is performed [49, 50].

The findings of the present review were further limited by the high risk of within-study bias observed in many studies, caused primarily by a high risk of bias in measurement of the outcome, and consequently resulting in a lowering of the confidence in several effect estimates. The reason for this grading was due to many studies not blinding participants to the provision of the pre- and/or post-exercise standardised meals. Knowledge of meal consumption (or omission) is likely to influence subsequent energy intake and subjective hunger (but not energy expenditure or gastrointestinal hormone release). As standardised meals were often given as whole foods, this precludes the blinding of any meal provided, making comparisons between FastEx + NoMeal (no standardised meals) to both forms of fed exercise (1–2 standardised meals) particularly problematic. This issue can however be addressed using two approaches: (i) comparing a liquid meal in an opaque bottle to an energy-free, taste-matched liquid control, (ii) comparing FastEx + Meal (1 standardised meal) to FedEx + NoMeal (1 standardised meal), in which the standardised meal given after fasted exercise is identical to the standardised meal given before fed exercise. While this knowledge of meal provision may have contributed to the observed significant increase in subjective hunger between fasted and fed exercise interventions, it did not significantly increase 24-h energy intake. Indeed, the effect on 24-h energy intake occurred in the opposite direction to that predicted, suggesting that effect estimates for 24-h energy intake may be underestimated.

In summary, FastEx + NoMeal appears to produce the greatest reduction in short-term energy intake while also exhibiting an increase in subjective hunger and decrease in energy expenditure relative to fed exercise interventions. Future work should now investigate the effect of fasted exercise relative to fed exercise in populations where effects would be beneficial (e.g. individuals with obesity) and measure gastrointestinal hormone release where possible. Furthermore, these studies should look to prioritise comparisons between FastEx + Meal and FedEx + NoMeal in order to minimise measurement bias and to isolate the effect of fasted exercise itself. This information would help to develop evidence-based integrated diet-exercise guidelines that can best promote improvements in energy balance to support weight management.

## Supplementary information

Supplementary Appendix S1

Supplementary Appendix S2

Supplementary Appendix S3

Supplementary Appendix S4

Supplementary Appendix S5

Supplementary Appendix S6

Supplementary figure legends

Supplementary Material

Supplementary Material

Supplementary Material

Supplementary Material

Supplementary Material

Supplementary Material

Supplementary Material

Supplementary Material

Supplementary Material

Supplementary Material

Supplementary Material

Supplementary Material

## Data Availability

Please contact the corresponding author for data requests.
