# Supplementary material for: The acute effect of fasted exercise on energy intake, energy expenditure, subjective hunger and gastrointestinal hormone release compared to fed exercise in healthy individuals: a systematic review and network meta-analysis
Source: Int J Obes (Lond). 2021 Nov 3;46(2):255–68. doi: 10.1038/s41366-021-00993-1 (PMC8794783; doi:10.1038/s41366-021-00993-1)
Supplement: Supplementary file 1 — Supplementary Appendix S1 [file 41366_2021_993_MOESM1_ESM.docx]

**Supplementary Appendix S1:** Database search strategies

EMBASE

*Searched:* 07/07/2020

"((carbohydrate or breakfast or postprandial or fed or feeding or fast or fasted or fasting or skipping or omission or low glycogen or glycogen depletion or carbohydrate loading or glucose deprived or low glucose) and (Exercise or exercising or physical activity or walk or walking or run or running or cycle or cycling) and (Crossover or cross-over or counterbalanced or acute or randomized or randomised) and (Appetite or energy or glucagon-like peptide 1 or glp-1 or glp1 or peptide tyrosine tyrosine or peptide YY or PYY or ghrelin)).ab"

MEDLINE

*Searched:* 11/07/2020

"((carbohydrate or breakfast or postprandial or fed or feeding or fast or fasted or fasting or skipping or omission or low glycogen or glycogen depletion or carbohydrate loading or glucose deprived or low glucose) and (Exercise or exercising or physical activity or walk or walking or run or running or cycle or cycling) and (Crossover or cross-over or counterbalanced or acute or randomized or randomised) and (Appetite or energy or glucagon-like peptide 1 or glp-1 or glp1 or peptide tyrosine tyrosine or peptide YY or PYY or ghrelin)).ab"

PsycInfo

*Searched:* 07/07/2020

"((carbohydrate or breakfast or postprandial or fed or feeding or fast or fasted or fasting or skipping or omission or low glycogen or glycogen depletion or carbohydrate loading or glucose deprived or low glucose) and (Exercise or exercising or physical activity or walk or walking or run or running or cycle or cycling) and (Crossover or cross-over or counterbalanced or acute or randomized or randomised) and (Appetite or energy or glucagon-like peptide 1 or glp-1 or glp1 or peptide tyrosine tyrosine or peptide YY or PYY or ghrelin)).ab"

PubMed

*Searched:* 06/07/2020

"(((carbohydrate OR breakfast OR postprandial OR fed OR feeding OR fast OR fasted OR fasting OR skipping OR omission OR low glycogen OR glycogen depletion OR carbohydrate loading OR glucose deprived OR low glucose) AND(Exercise OR exercising OR physical activity OR walk OR walking OR run OR running OR cycle OR cycling)) AND (Crossover OR cross-over OR counterbalanced OR acute OR randomized OR randomised)) AND (Appetite OR energy OR glucagon-like peptide 1 OR glp-1 OR glp1 OR peptide tyrosine tyrosine OR peptide YY OR PYY OR ghrelin)"

Scopus

*Searched:* 10/07/2020

"(TITLE-ABS-KEY(carbohydrate) OR TITLE-ABS-KEY(breakfast) OR TITLE-ABS-KEY(postprandial) OR TITLE-ABS-KEY(fed) OR TITLE-ABS-KEY(feeding) OR TITLE-ABS-KEY(fast) OR TITLE-ABS-KEY(fasted) OR TITLE-ABS-KEY(fasting) OR TITLE-ABS-KEY(skipping) OR TITLE-ABS-KEY(omission) OR TITLE-ABS-KEY(low glycogen) OR TITLE-ABS-KEY(glycogen depletion) OR TITLE-ABS-KEY(carbohydrate loading) OR TITLE-ABS-KEY(glucose deprived) OR TITLE-ABS-KEY(low glucose) AND TITLE-ABS-KEY(exercise) OR TITLE-ABS-KEY(exercising) OR TITLE-ABS-KEY(physical activity) OR TITLE-ABS-KEY(walk) OR TITLE-ABS-KEY(walking) OR TITLE-ABS-KEY(run) OR TITLE-ABS-KEY(running) OR TITLE-ABS-KEY(cycle) OR TITLE-ABS-KEY(cycling) AND TITLE-ABS-KEY(crossover) OR TITLE-ABS-KEY(cross-over) OR TITLE-ABS-KEY(counterbalanced) OR TITLE-ABS-KEY(acute) OR TITLE-ABS-KEY(randomised) OR TITLE-ABS-KEY(randomized) AND TITLE-ABS-KEY(appetite) OR TITLE-ABS-KEY(energy) OR TITLE-ABS-KEY(glucagon-like peptide 1) OR TITLE-ABS-KEY(glp-1) OR TITLE-ABS-KEY(glp1) OR TITLE-ABS-KEY(peptide tyrosine tyrosine) OR TITLE-ABS-KEY(peptide YY) OR TITLE-ABS-KEY(PYY) OR TITLE-ABS-KEY(ghrelin))"

The Cochrane Library (CENTRAL)

*Searched:* 07/07/2020

"(((carbohydrate OR breakfast OR postprandial OR fed OR feeding OR fast OR fasted OR fasting OR skipping OR omission OR low glycogen OR glycogen depletion OR carbohydrate loading OR glucose deprived OR low glucose) AND (Exercise OR exercising OR physical activity OR walk OR walking OR run OR running OR cycle OR cycling)) AND (Crossover OR cross-over OR counterbalanced OR acute OR randomized OR randomised)) AND (Appetite OR energy OR glucagon-like peptide 1 OR glp-1 OR glp1 OR peptide tyrosine tyrosine OR peptide YY OR PYY OR ghrelin)"

Web of Science

*Searched:* 07/07/2020

"TOPIC: (carbohydrate OR breakfast OR postprandial OR fed OR feeding OR fast OR fasted OR fasting OR skipping OR omission OR low glycogen OR glycogen depletion OR carbohydrate loading OR glucose deprived OR low glucose) AND TOPIC: (Exercise OR exercising OR physical activity OR walk OR walking OR run OR running OR cycle OR cycling) AND TOPIC: (Crossover OR cross-over OR counterbalanced OR acute OR randomized OR randomised) AND TOPIC: (Appetite OR energy OR glucagon-like peptide 1 OR glp-1 OR glp1 OR peptide tyrosine tyrosine OR peptide YY OR PYY OR ghrelin)"
