# Supplementary material for: The acute effect of fasted exercise on energy intake, energy expenditure, subjective hunger and gastrointestinal hormone release compared to fed exercise in healthy individuals: a systematic review and network meta-analysis
Source: Int J Obes (Lond). 2021 Nov 3;46(2):255–68. doi: 10.1038/s41366-021-00993-1 (PMC8794783; doi:10.1038/s41366-021-00993-1)
Supplement: Supplementary file 2 — Supplementary Appendix S2 [file 41366_2021_993_MOESM2_ESM.docx]

**Supplementary Appendix S2:** Risk of bias tables

*Ad libitum* meal energy intake:

|  | **(1)** | **(2)** | **(3)** | **(4)** | **(5)** | **(6)** |
| --- | --- | --- | --- | --- | --- | --- |
| Bachman *et al.,* 2016 | **L** | **L** | **L** | **H** | **L** | **H** |
| Brown *et al.,* 2016 | **U** | **L** | **L** | **L** | **L** | **U** |
| Clayton *et al.,* 2015 | **U** | **L** | **L** | **L** | **L** | **U** |
| Edinburgh *et al.,* 2019 | **L** | **L** | **L** | **H** | **L** | **H** |
| Farah & Gill, 2013 | **L** | **L** | **L** | **U** | **L** | **U** |
| Gonzalez *et al.,* 2013 | **U** | **L** | **L** | **H** | **L** | **H** |
| Griffiths *et al.,* 2020^a^ | **U** | **L** | **L** | **H** | **L** | **H** |
| Griffiths *et al.,* 2020^b^ | **U** | **L** | **L** | **H** | **L** | **H** |
| Hunschede *et al.,* 2015 | **U** | **L** | **L** | **L** | **L** | **U** |
| Tamam *et al.,* 2012^a^ | **U** | **L** | **L** | **L** | **L** | **U** |
| Tamam *et al.,* 2012^b^ | **U** | **L** | **L** | **L** | **L** | **U** |
| Tamam *et al.,* 2012^c^ | **U** | **L** | **L** | **L** | **L** | **U** |
| Thivel *et al.,* 2020 | **U** | **L** | **L** | **H** | **L** | **H** |
| Veasey *et al.,* 2015 | **U** | **L** | **L** | **H** | **L** | **H** |

Within-lab energy intake:

|  | **(1)** | **(2)** | **(3)** | **(4)** | **(5)** | **(6)** |
| --- | --- | --- | --- | --- | --- | --- |
| Bachman *et al.,* 2016 | **L** | **L** | **L** | **H** | **L** | **H** |
| Brown *et al.,* 2016 | **U** | **L** | **L** | **L** | **L** | **U** |
| Clayton *et al.,* 2015 | **U** | **L** | **L** | **L** | **L** | **U** |
| Edinburgh *et al.,* 2019 | **L** | **L** | **L** | **H** | **L** | **H** |
| Farah & Gill, 2013 | **L** | **L** | **L** | **U** | **L** | **U** |
| Gonzalez *et al.,* 2013 | **U** | **L** | **L** | **H** | **L** | **H** |
| Griffiths *et al.,* 2020^a^ | **U** | **L** | **L** | **H** | **L** | **H** |
| Griffiths *et al.,* 2020^b^ | **U** | **L** | **L** | **H** | **L** | **H** |
| Hunschede *et al.,* 2015 | **U** | **L** | **L** | **L** | **L** | **U** |
| Tamam *et al.,* 2012^a^ | **U** | **L** | **L** | **L** | **L** | **U** |
| Tamam *et al.,* 2012^b^ | **U** | **L** | **L** | **L** | **L** | **U** |
| Tamam *et al.,* 2012^c^ | **U** | **L** | **L** | **L** | **L** | **U** |
| Thivel *et al.,* 2020 | **U** | **L** | **L** | **H** | **L** | **H** |
| Veasey *et al.,* 2015 | **U** | **L** | **L** | **H** | **L** | **H** |

24-hour energy intake:

|  | **(1)** | **(2)** | **(3)** | **(4)** | **(5)** | **(6)** |
| --- | --- | --- | --- | --- | --- | --- |
| Bachman *et al.,* 2016 | **L** | **L** | **L** | **H** | **L** | **H** |
| Broad *et al.,* 2020 | **U** | **L** | **L** | **U** | **L** | **U** |
| Brown *et al.,* 2016 | **U** | **L** | **L** | **L** | **L** | **U** |
| Edinburgh *et al.,* 2019 | **L** | **L** | **L** | **H** | **L** | **H** |
| McIver *et al.,* 2019 | **U** | **L** | **L** | **H** | **L** | **H** |
| Veasey *et al.,* 2015 | **U** | **L** | **L** | **H** | **L** | **H** |

Energy expenditure:

|  | **(1)** | **(2)** | **(3)** | **(4)** | **(5)** | **(6)** |
| --- | --- | --- | --- | --- | --- | --- |
| Bennard & Doucet, 2006a | **?** | **+** | **+** | **+** | **+** | **U** |
| Bennard & Doucet, 2006b | **?** | **+** | **+** | **+** | **+** | **U** |
| Broad et al., 2020 | **?** | **+** | **+** | **+** | **+** | **U** |
| Davis et al., 1989a | **?** | **+** | **+** | **+** | **+** | **U** |
| Davis et al., 1989b | **?** | **+** | **+** | **+** | **+** | **U** |
| Davis et al., 1989c | **?** | **+** | **+** | **+** | **+** | **U** |
| Edinburgh et al., 2019 | **+** | **+** | **+** | **+** | **+** | **L** |
| Farah & Gill, 2013 | **+** | **+** | **+** | **+** | **+** | **L** |

Subjective hunger:

|  | **(1)** | **(2)** | **(3)** | **(4)** | **(5)** | **(6)** |
| --- | --- | --- | --- | --- | --- | --- |
| Broad *et al.,* 2020 | **U** | **L** | **L** | **U** | **L** | **U** |
| Brown *et al.,* 2016 | **U** | **L** | **L** | **L** | **L** | **U** |
| Cheng *et al.,* 2009 | **U** | **L** | **L** | **L** | **L** | **U** |
| Farah & Gill, 2013 | **L** | **L** | **L** | **U** | **L** | **U** |
| Gonzalez *et al.,* 2013 | **U** | **L** | **L** | **H** | **L** | **H** |
| Griffiths *et al.,* 2020^a^ | **U** | **L** | **L** | **H** | **L** | **H** |
| Griffiths *et al.,* 2020^b^ | **U** | **L** | **L** | **H** | **L** | **H** |
| McIver *et al.,* 2019 | **U** | **L** | **L** | **H** | **L** | **H** |
| McIver *et al.,* 2019 | **U** | **L** | **L** | **H** | **L** | **H** |
| Thivel *et al.,* 2020 | **U** | **L** | **L** | **H** | **L** | **H** |
| Veasey *et al.,* 2015 | **U** | **L** | **L** | **H** | **L** | **H** |

Acyl-ghrelin:

|  | **(1)** | **(2)** | **(3)** | **(4)** | **(5)** | **(6)** |
| --- | --- | --- | --- | --- | --- | --- |
| Griffiths *et al.,* 2020^a^ | **U** | **L** | **L** | **L** | **L** | **U** |
| Griffiths *et al.,* 2020^b^ | **U** | **L** | **L** | **L** | **L** | **U** |
| McIver *et al.,* 2019 | **U** | **L** | **L** | **L** | **L** | **U** |

Glucagon-like peptide 1:

|  | **(1)** | **(2)** | **(3)** | **(4)** | **(5)** | **(6)** |
| --- | --- | --- | --- | --- | --- | --- |
| Brown *et al.,* 2016 | **U** | **+** | **+** | **+** | **+** | **U** |
| Gonzalez *et al.,* 2013 | **U** | **+** | **+** | **+** | **+** | **U** |
| McIver *et al.,* 2019 | **U** | **+** | **+** | **+** | **+** | **U** |

Peptide YY:

|  | **(1)** | **(2)** | **(3)** | **(4)** | **(5)** | **(6)** |
| --- | --- | --- | --- | --- | --- | --- |
| Cheng *et al.,* 2009 | **U** | **L** | **L** | **L** | **L** | **U** |
| McIver *et al.,* 2019 | **U** | **L** | **L** | **L** | **L** | **U** |

(1) Bias arising from the randomization process; (2) Bias due to deviations from intended interventions; (3) Bias due to missing outcome data; (4) Bias in measurement of the outcome; (5) Bias in the selection of the reported result (6) Overall risk of bias; [H] high risk of bias; [?] unclear risk of bias; [L] low risk of bias.
