# Supplementary material for: The acute effect of fasted exercise on energy intake, energy expenditure, subjective hunger and gastrointestinal hormone release compared to fed exercise in healthy individuals: a systematic review and network meta-analysis
Source: Int J Obes (Lond). 2021 Nov 3;46(2):255–68. doi: 10.1038/s41366-021-00993-1 (PMC8794783; doi:10.1038/s41366-021-00993-1)
Supplement: Supplementary file 3 — Supplementary Appendix S3 [file 41366_2021_993_MOESM3_ESM.docx]

**Supplementary Appendix S3:** Intervention ranks based on P-scores

*Ad libitum* meal energy intake:

| **Intervention** | **P-score** | **Rank** |
| --- | --- | --- |
| FedEx+Meal | 0.970 | 1 |
| FedEx+NoMeal | 0.473 | 2 |
| FastEx+Meal | 0.445 | 3 |
| FastEx+NoMeal | 0.112 | 4 |

Within-lab energy intake:

| **Intervention** | **P-score** | **Rank** |
| --- | --- | --- |
| FastEx+NoMeal | 0.990 | 1 |
| FedEx+NoMeal | 0.452 | 2 |
| FastEx+Meal | 0.451 | 3 |
| FedEx+Meal | 0.107 | 4 |

24-hour energy intake:

| **Intervention** | **P-score** | **Rank** |
| --- | --- | --- |
| FastEx+NoMeal | 0.964 | 1 |
| FastEx+Meal | 0.501 | 2 |
| FedEx+NoMeal | 0.403 | 3 |
| FedEx+Meal | 0.132 | 4 |

Energy expenditure:

| **Intervention** | **P-score** | **Rank** |
| --- | --- | --- |
| FastEx+Meal | 0.744 | 1 |
| FedEx+Meal | 0.683 | 2 |
| FedEx+NoMeal | 0.561 | 3 |
| FastEx+NoMeal | 0.013 | 4 |

Subjective hunger:

| **Intervention** | **P-score** | **Rank** |
| --- | --- | --- |
| FedEx+Meal | 0.980 | 1 |
| FedEx+NoMeal | 0.686 | 2 |
| FastEx+Meal | 0.323 | 3 |
| FastEx+NoMeal | 0.010 | 4 |
