# Supplementary material for: The acute effect of fasted exercise on energy intake, energy expenditure, subjective hunger and gastrointestinal hormone release compared to fed exercise in healthy individuals: a systematic review and network meta-analysis
Source: Int J Obes (Lond). 2021 Nov 3;46(2):255–68. doi: 10.1038/s41366-021-00993-1 (PMC8794783; doi:10.1038/s41366-021-00993-1)
Supplement: Supplementary file 4 — Supplementary Appendix S4 [file 41366_2021_993_MOESM4_ESM.docx]

**Supplementary Appendix S4:** League tables of results

*Ad libitum* meal energy intake (kJ):

| **FastEx+Meal** | - | 431 (-370 to 1232) P=0.292 | 138 (-1001 to 1277) P=0.812 |
| --- | --- | --- | --- |
| -328*  (-1201 to 545) P=0.461 | **FastEx+NoMeal** | - | 335  (-173 to 843) P=0.196 |
| 496 (-173 to 1165) P=0.146 | 824*  (172 to 1477) P=0.013 | **FedEx+Meal** | -507 (-936 to -79) P=0.020 |
| 7 (-703 to 717) P=0.985 | 335 (-173 to 843) P=0.196 | -489 (-898 to -80) P=0.019 | **FedEx+NoMeal** |

Within-lab energy intake (kJ):

| **FastEx+Meal** | - | -520 (-1738 to 698) P=0.403 | 138 (-1556 to 1832) P=0.873 |
| --- | --- | --- | --- |
| 1287*  (-39 to 2613) P=0.057 | **FastEx+NoMeal** | - | -1326  (-2102 to -550) P=0.001 |
| -428 (-1441 to 584) P=0.407 | -1715*  (-2721 to -710) P=0.001 | **FedEx+Meal** | 362 (-310 to 1033) P=0.291 |
| -39 (-1114 to 1036) P=0.944 | -1326 (-2102 to -550) P=0.001 | 389 (-250 to 1029) P=0.232 | **FedEx+NoMeal** |

24-hour energy intake (kJ):

| **FastEx+Meal** | - | -699 (-2381 to 983) P=0.415 | -521 (-3202 to 2160) P=0.703 |
| --- | --- | --- | --- |
| 1934*  (-736 to 4605) P=0.156 | **FastEx+NoMeal** | - | -2095 (-3910 to -280) P=0.024 |
| -841 (-2361 to 679) 0.278 | -2775*  (-5383 to -166) P=0.037 | **FedEx+Meal** | 950 (-1374 to 3274) P=0.423 |
| -161 (-2120 to 1798) P=0.872 | -2095  (-3910 to -280) P=0.024 | 680 (-1194 to 2553) 0.477 | **FedEx+NoMeal** |

Energy expenditure (kJ/min):

| **FastEx+Meal** | 0.58  (0.10 to 1.07) P=0.019 | -0.00  (-0.70 to 0.69) P=0.992 | 0.07  (-0.15 to 0.30) P=0.529 |
| --- | --- | --- | --- |
| 0.74  (0.31 to 1.18) P<0.001 | **FastEx+NoMeal** | - | -0.83  (-1.31 to -0.35) P<0.001 |
| -0.00  (-0.70 to 0.69) P=0.992 | -0.75*  (-1.57 to 0.08) P=0.075 | **FedEx+Meal** | - |
| 0.07  (-0.15 to 0.30) P=0.529 | -0.67  (-1.10 to -0.23) P=0.003 | 0.08*  (-0.66 to 0.81) P=0.837 | **FedEx+NoMeal** |

Subjective hunger (mm):

| **FastEx+Meal** | - | 20  (11 to 28) P<0.001 | 12 (2 to 21) P=0.016 |
| --- | --- | --- | --- |
| -10*  (-20 to 0) P=0.062 | **FastEx+NoMeal** | - | 23 (16 to 30) P<0.001 |
| 19 (12 to 26) P<0.001 | 29*  (18 to 39) P<0.001 | **FedEx+Meal** | -5 (-15 to 5) 0.302 |
| 13  (5 to 21) P=0.001 | 23 (16 to 30) P<0.001 | -6 (-14 to 2) P=0.119 | **FedEx+NoMeal** |

Mean difference, (95% confidence intervals), and P values. Comparisons, column versus row, should be read from left to right. Values in the upper triangle are results from the pairwise meta-analysis and values from the bottom triangle are results from the network meta-analysis. *denotes effect estimates and confidence intervals calculated using indirect evidence only. Highlighted boxes denote a statistically significant difference (P<0.05).
