# Supplementary material for: The acute effect of fasted exercise on energy intake, energy expenditure, subjective hunger and gastrointestinal hormone release compared to fed exercise in healthy individuals: a systematic review and network meta-analysis
Source: Int J Obes (Lond). 2021 Nov 3;46(2):255–68. doi: 10.1038/s41366-021-00993-1 (PMC8794783; doi:10.1038/s41366-021-00993-1)
Supplement: Supplementary file 5 — Supplementary Appendix S5 [file 41366_2021_993_MOESM5_ESM.docx]

**Supplementary Appendix S5:** Results from SIDE approach

*Ad libitum* meal energy intake (kJ):

| **Comparison** | **Number of studies providing direct evidence** | **Estimated effect derived from direct evidence** | **Estimated effect derived from indirect evidence** | **Difference between direct and indirect treatment estimates** | **P value of test for disagreement** |
| --- | --- | --- | --- | --- | --- |
| FastEx+Meal  vs FastEx+NoMeal | 0 | - | -328 | - | - |
| FastEx+Meal  vs  FedEx+Meal | 2 | 431 | 645 | -214 | 0.773 |
| FastEx+Meal  vs  FedEx+NoMeal | 1 | 138 | -76 | 214 | 0.773 |
| FastEx+NoMeal  vs  FedEx+Meal | 0 | - | 824 | - | - |
| FastEx+NoMeal  vs  FedEx+NoMeal | 5 | 335 | - | - | - |
| FedEx+Meal  vs  FedEx+NoMeal | 6 | -507 | -293 | -214 | 0.773 |

Within-lab energy intake (kJ):

| **Comparison** | **Number of studies providing direct evidence** | **Estimated effect derived from direct evidence** | **Estimated effect derived from indirect evidence** | **Difference between direct and indirect treatment estimates** | **P value of test for disagreement** |
| --- | --- | --- | --- | --- | --- |
| FastEx+Meal  vs FastEx+NoMeal | 0 | - | 1287 | - | - |
| FastEx+Meal  vs  FedEx+Meal | 2 | -520 | -224 | -296 | 0.791 |
| FastEx+Meal  vs  FedEx+NoMeal | 1 | 138 | -158 | 296 | 0.791 |
| FastEx+NoMeal  vs  FedEx+Meal | 0 | - | -1715 | - | - |
| FastEx+NoMeal  vs  FedEx+NoMeal | 5 | -1326 | - | - | - |
| FedEx+Meal  vs  FedEx+NoMeal | 6 | 362 | 658 | -296 | 0.791 |

24-hour energy intake (kJ):

| **Comparison** | **Number of studies providing direct evidence** | **Estimated effect derived from direct evidence** | **Estimated effect derived from indirect evidence** | **Difference between direct and indirect treatment estimates** | **P value of test for disagreement** |
| --- | --- | --- | --- | --- | --- |
| FastEx+Meal  vs FastEx+NoMeal | 0 | - | 1934 | - | - |
| FastEx+Meal  vs  FedEx+Meal | 2 | -699 | -1471 | 772 | 0.700 |
| FastEx+Meal  vs  FedEx+NoMeal | 1 | -521 | 251 | -772 | 0.700 |
| FastEx+NoMeal  vs  FedEx+Meal | 0 | - | -27745 | - | - |
| FastEx+NoMeal  vs  FedEx+NoMeal | 2 | -2095 | - | - | - |
| FedEx+Meal  vs  FedEx+NoMeal | 1 | 950 | 178 | 772 | 0.700 |

Energy expenditure (kJ/min):

| **Comparison** | **Number of studies providing direct evidence** | **Estimated effect derived from direct evidence** | **Estimated effect derived from indirect evidence** | **Difference between direct and indirect treatment estimates** | **P value of test for disagreement** |
| --- | --- | --- | --- | --- | --- |
| FastEx+Meal  vs FastEx+NoMeal | 1 | 0.58 | 1.43 | -0.85 | 0.135 |
| FastEx+Meal  vs  FedEx+Meal | 1 | -0.00 | - | - | - |
| FastEx+Meal  vs  FedEx+NoMeal | 5 | 0.07 | - | - | - |
| FastEx+NoMeal  vs  FedEx+Meal | 0 | - | -0.75 | - | - |
| FastEx+NoMeal  vs  FedEx+NoMeal | 1 | -0.83 | 0.02 | -0.85 | 0.135 |
| FedEx+Meal  vs  FedEx+NoMeal | 0 | - | 0.08 | - | - |

Subjective hunger (mm):

| **Comparison** | **Number of studies providing direct evidence** | **Estimated effect derived from direct evidence** | **Estimated effect derived from indirect evidence** | **Difference between direct and indirect treatment estimates** | **P value of test for disagreement** |
| --- | --- | --- | --- | --- | --- |
| FastEx+Meal  vs FastEx+NoMeal | 0 | - | -10 | - | - |
| FastEx+Meal  vs  FedEx+Meal | 3 | 19 | 17 | 3 | 0.730 |
| FastEx+Meal  vs  FedEx+NoMeal | 2 | 12 | 15 | -3 | 0.730 |
| FastEx+NoMeal  vs  FedEx+Meal | 0 | - | 29 | - | - |
| FastEx+NoMeal  vs  FedEx+NoMeal | 4 | 23 | - | - | - |
| FedEx+Meal  vs  FedEx+NoMeal | 2 | -5 | -8 | 3 | 0.730 |
