# Supplementary material for: The acute effect of fasted exercise on energy intake, energy expenditure, subjective hunger and gastrointestinal hormone release compared to fed exercise in healthy individuals: a systematic review and network meta-analysis
Source: Int J Obes (Lond). 2021 Nov 3;46(2):255–68. doi: 10.1038/s41366-021-00993-1 (PMC8794783; doi:10.1038/s41366-021-00993-1)
Supplement: Supplementary file 6 — Supplementary Appendix S6 [file 41366_2021_993_MOESM6_ESM.docx]

**Supplementary Appendix S6:** Results from the confidence in evidence evaluation using the CINeMA framework

For all effect estimates, concerns regarding within-study bias were thought to drive the increase in imprecision and/or heterogeneity. Confidence was therefore downgraded by two levels for comparisons that displayed ‘major concerns’ or ‘some concerns’ for within-study bias, and ‘major concerns’ or ‘some concerns’ for imprecision and/or heterogeneity Similarly, confidence was downgraded by one level for comparisons that displayed ‘some concerns’ for within-study bias, and ‘some concerns’ for imprecision and/or heterogeneity

*Ad libitum* meal energy intake:

| **Comparison** | **Number of studies** | **Within-study bias** | **Reporting bias** | **Indirectness** | **Imprecision** | **Heterogeneity** | **Incoherence** | **Confidence rating** |
| --- | --- | --- | --- | --- | --- | --- | --- | --- |
| FastEx+Meal  vs  FedEx+Meal | 2 | Major concerns | Undetected | No concerns | Some concerns | Some concerns | No concerns | Low |
| FastEx+Meal  vs  FedEx+NoMeal | 1 | Some concerns | Undetected | No concerns | Major concerns | No concerns | No concerns | Low |
| FastEx+NoMeal  vs  FedEx+NoMeal | 5 | Major concerns | Undetected | No concerns | Some concerns | Some concerns | No concerns | Low |
| FedEx+Meal  vs  FedEx+NoMeal | 6 | Some concerns | Undetected | No concerns | No concerns | Major concerns | No concerns | Low |
| FastEx+Meal  vs  FastEx+NoMeal | 0 | Major concerns | Undetected | No concerns | Major concerns | No concerns | No concerns | Low |
| FastEx+NoMeal  vs  FedEx+Meal | 0 | Some concerns | Undetected | No concerns | No concerns | Some concerns | No concerns | Moderate |

Effect estimates for FastEx+Meal vs FedEx+NoMeal, FastEx+NoMeal vs FedEx+NoMeal, and FedEx+NoMeal vs FedEx+Meal were classified as low confidence. FastEx+Meal vs FedEx+Meal and FastEx+Meal vs FastEx+NoMeal effect estimates were also classified as low confidence, whereas the effect estimate for FastEx+NoMeal vs FedEx+Meal was classified as moderate confidence.

Within-lab energy intake:

| **Comparison** | **Number of studies** | **Within-study bias** | **Reporting bias** | **Indirectness** | **Imprecision** | **Heterogeneity** | **Incoherence** | **Confidence rating** |
| --- | --- | --- | --- | --- | --- | --- | --- | --- |
| FastEx+Meal  vs  FedEx+Meal | 2 | Major concerns | Undetected | No concerns | Major concerns | No concerns | No concerns | Low |
| FastEx+Meal  vs  FedEx+NoMeal | 1 | Some concerns | Undetected | No concerns | Major concerns | No concerns | No concerns | Low |
| FastEx+NoMeal  vs  FedEx+NoMeal | 5 | Major concerns | Undetected | No concerns | No concerns | Major concerns | No concerns | Low |
| FedEx+Meal  vs  FedEx+NoMeal | 6 | Some concerns | Undetected | No concerns | Some concerns | Some concerns | No concerns | Moderate |
| FastEx+Meal  vs  FastEx+NoMeal | 0 | Major concerns | Undetected | No concerns | Some concerns | Some concerns | No concerns | Low |
| FastEx+NoMeal  vs  FedEx+Meal | 0 | Some concerns | Undetected | No concerns | No concerns | Some concerns | No concerns | Moderate |

Effect estimates for FastEx+Meal vs FedEx+NoMeal, FastEx+NoMeal vs FedEx+NoMeal, and FedEx+NoMeal vs FedEx+Meal were classified as low, low, moderate confidence, respectively. FastEx+Meal vs FedEx+Meal and FastEx+Meal vs FastEx+NoMeal effect estimates were also classified as low confidence, whereas the effect estimate for FastEx+NoMeal vs FedEx+Meal was classified as moderate confidence.

24-hour energy intake:

| **Comparison** | **Number of studies** | **Within-study bias** | **Reporting bias** | **Indirectness** | **Imprecision** | **Heterogeneity** | **Incoherence** | **Confidence rating** |
| --- | --- | --- | --- | --- | --- | --- | --- | --- |
| FastEx+Meal  vs  FedEx+Meal | 2 | Major concerns | Undetected | No concerns | Some concerns | Some concerns | No concerns | Low |
| FastEx+Meal  vs  FedEx+NoMeal | 1 | Some concerns | Undetected | No concerns | Major concerns | No concerns | No concerns | Low |
| FastEx+NoMeal  vs  FedEx+NoMeal | 2 | Major concerns | Undetected | No concerns | No concerns | Major concerns | No concerns | Low |
| FedEx+Meal  vs  FedEx+NoMeal | 1 | Some concerns | Undetected | No concerns | Major concerns | No concerns | No concerns | Low |
| FastEx+Meal  vs  FastEx+NoMeal | 0 | Major concerns | Undetected | No concerns | Some concerns | Some concerns | No concerns | Low |
| FastEx+NoMeal  vs  FedEx+Meal | 0 | Major concerns | Undetected | No concerns | No concerns | Major concerns | No concerns | Low |

All effect estimates for all comparisons were classified as low confidence.

Energy expenditure:

| **Comparison** | **Number of studies** | **Within-study bias** | **Reporting bias** | **Indirectness** | **Imprecision** | **Heterogeneity** | **Incoherence** | **Confidence rating** |
| --- | --- | --- | --- | --- | --- | --- | --- | --- |
| FastEx+Meal  vs  FastEx+NoMeal | 1 | Some concerns | Undetected | No concerns | No concerns | No concerns | No concerns | Moderate |
| FastEx+Meal  vs  FedEx+Meal | 1 | No concerns | Undetected | No concerns | No concerns | No concerns | No concerns | High |
| FastEx+Meal  vs  FedEx+NoMeal | 5 | Some concerns | Undetected | No concerns | No concerns | No concerns | No concerns | Moderate |
| FastEx+NoMeal  vs  FedEx+NoMeal | 1 | Some concerns | Undetected | No concerns | No concerns | No concerns | No concerns | Moderate |
| FastEx+NoMeal  vs  FedEx+Meal | 0 | Some concerns | Undetected | No concerns | No concerns | No concerns | No concerns | Moderate |
| FedEx+Meal  vs  FedEx+NoMeal | 0 | No concerns | Undetected | No concerns | No concerns | No concerns | No concerns | High |

Effect estimates for FastEx+Meal vs FedEx+NoMeal, FastEx+NoMeal vs FedEx+NoMeal, and FedEx+NoMeal vs FedEx+Meal were classified as moderate, moderate, and high confidence, respectively. FastEx+Meal vs FastEx+NoMeal and FastEx+NoMeal vs FedEx+Meal effect estimates were classified as moderate confidence, and the effect estimate for FastEx+Meal vs FedEx+Meal was classified as high confidence.

Subjective hunger:

| **Comparison** | **Number of studies** | **Within-study bias** | **Reporting bias** | **Indirectness** | **Imprecision** | **Heterogeneity** | **Incoherence** | **Confidence rating** |
| --- | --- | --- | --- | --- | --- | --- | --- | --- |
| FastEx+Meal  vs  FedEx+Meal | 3 | Major concerns | Undetected | No concerns | No concerns | No concerns | No concerns | Low |
| FastEx+Meal  vs  FedEx+NoMeal | 2 | Some concerns | Undetected | No concerns | No concerns | Some concerns | No concerns | Moderate |
| FastEx+NoMeal  vs  FedEx+NoMeal | 4 | Major concerns | Undetected | No concerns | No concerns | No concerns | No concerns | Low |
| FedEx+Meal  vs  FedEx+NoMeal | 2 | Major concerns | Undetected | No concerns | Some concerns | Some concerns | No concerns | Low |
| FastEx+Meal  vs  FastEx+NoMeal | 0 | Major concerns | Undetected | No concerns | Some concerns | No concerns | No concerns | Low |
| FastEx+NoMeal  vs  FedEx+Meal | 0 | Major concerns | Undetected | No concerns | No concerns | No concerns | No concerns | Low |

Effect estimates for FastEx+Meal vs FedEx+NoMeal, FastEx+NoMeal vs FedEx+NoMeal, and FedEx+NoMeal vs FedEx+Meal were classified as moderate, low, and low confidence, respectively. FastEx+Meal vs FastEx+NoMeal, FastEx+NoMeal vs FedEx+Meal and FastEx+Meal vs FedEx+Meal were classified as low confidence.
