# Supplementary material for: The acute effect of fasted exercise on energy intake, energy expenditure, subjective hunger and gastrointestinal hormone release compared to fed exercise in healthy individuals: a systematic review and network meta-analysis
Source: Int J Obes (Lond). 2021 Nov 3;46(2):255–68. doi: 10.1038/s41366-021-00993-1 (PMC8794783; doi:10.1038/s41366-021-00993-1)
Supplement: Supplementary file 7 — Supplementary figure legends [file 41366_2021_993_MOESM7_ESM.docx]

**Figure S1:** Risk of bias summary for A) *ad libitum* meal energy intake, B) within-lab energy intake, C) 24-hour energy intake, D) energy expenditure, and E) subjective hunger.

**Figure S2:** Risk of bias summary for A) acyl-ghrelin, B) glucagon-like peptide 1, and C) peptide YY.

**Figure S3:** Network graphs for A) *ad libitum* meal energy intake, B) within-lab energy intake, C) 24-hour energy intake, D) energy expenditure, and E) subjective hunger. Node size is proportional to the number of participants that undertook that intervention, and line thickness is proportional to the number of studies directly comparing the connected interventions. Numbers between nodes represent number of comparisons. Green nodes depict fed exercise interventions and blue nodes depict fasted exercise interventions. FastEx+Meal, fasted exercise with a standardised post-exercise meal; FastEx+NoMeal, fasted exercise without a standardised post-exercise meal; FedEx+Meal, fed exercise with a standardised post-exercise meal; FedEx+NoMeal, fed exercise without a standardised post-exercise meal.

**Figure S4:** Forest plot of individual study effect estimates for *ad libitum* energy intake. Data are presented as mean differences ± 95% confidence intervals. FastEx+Meal, fasted exercise with a standardised post-exercise meal; FastEx+NoMeal, fasted exercise without a standardised post-exercise meal; FedEx+Meal, fed exercise with a standardised post-exercise meal; FedEx+NoMeal, fed exercise without a standardised post-exercise meal.

**Figure S5:** Comparison-adjusted funnel plots for A) *ad libitum* meal energy intake, B) within-lab energy intake, C) 24-hour energy intake, D) energy expenditure, and E) subjective hunger. FastEx+Meal, fasted exercise with a standardised post-exercise meal; FastEx+NoMeal, fasted exercise without a standardised post-exercise meal; FedEx+Meal, fed exercise with a standardised post-exercise meal; FedEx+NoMeal, fed exercise without a standardised post-exercise meal.

**Figure S6:** Forest plot of individual study effect estimates for within-lab energy intake. Data are presented as mean differences ± 95% confidence intervals. FastEx+Meal, fasted exercise with a standardised post-exercise meal; FastEx+NoMeal, fasted exercise without a standardised post-exercise meal; FedEx+Meal, fed exercise with a standardised post-exercise meal; FedEx+NoMeal, fed exercise without a standardised post-exercise meal.

**Figure S7:** Forest plot of individual study effect estimates for 24-hour energy intake. Data are presented as mean differences ± 95% confidence intervals. FastEx+Meal, fasted exercise with a standardised post-exercise meal; FastEx+NoMeal, fasted exercise without a standardised post-exercise meal; FedEx+Meal, fed exercise with a standardised post-exercise meal; FedEx+NoMeal, fed exercise without a standardised post-exercise meal.

**Figure S8:** Forest plot of individual study effect estimates for energy expenditure. Data are presented as mean differences ± 95% confidence intervals. FastEx+Meal, fasted exercise with a standardised post-exercise meal; FastEx+NoMeal, fasted exercise without a standardised post-exercise meal; FedEx+Meal, fed exercise with a standardised post-exercise meal; FedEx+NoMeal, fed exercise without a standardised post-exercise meal.

**Figure S9:** Forest plot of individual study effect estimates for subjective hunger. Data are presented as mean differences ± 95% confidence intervals. FastEx+Meal, fasted exercise with a standardised post-exercise meal; FastEx+NoMeal, fasted exercise without a standardised post-exercise meal; FedEx+Meal, fed exercise with a standardised post-exercise meal; FedEx+NoMeal, fed exercise without a standardised post-exercise meal.

**Figure S10:** Forest plot of individual study effect estimates for acyl-ghrelin. Data are presented as mean differences ± 95% confidence intervals. FastEx+Meal, fasted exercise with a standardised post-exercise meal; FastEx+NoMeal, fasted exercise without a standardised post-exercise meal; FedEx+Meal, fed exercise with a standardised post-exercise meal; FedEx+NoMeal, fed exercise without a standardised post-exercise meal.

**Figure S11:** Forest plot of individual study effect estimates for glucagon-like peptide 1. Data are presented as mean differences ± 95% confidence intervals. FastEx+Meal, fasted exercise with a standardised post-exercise meal; FastEx+NoMeal, fasted exercise without a standardised post-exercise meal; FedEx+Meal, fed exercise with a standardised post-exercise meal; FedEx+NoMeal, fed exercise without a standardised post-exercise meal.

**Figure S12:** Forest plot of individual study effect estimates for peptide YY. Data are presented as mean differences ± 95% confidence intervals. FastEx+Meal, fasted exercise with a standardised post-exercise meal; FastEx+NoMeal, fasted exercise without a standardised post-exercise meal; FedEx+Meal, fed exercise with a standardised post-exercise meal; FedEx+NoMeal, fed exercise without a standardised post-exercise meal.
