# Supplementary figures and images for: The acute effect of fasted exercise on energy intake, energy expenditure, subjective hunger and gastrointestinal hormone release compared to fed exercise in healthy individuals: a systematic review and network meta-analysis
Source: Int J Obes (Lond). 2021 Nov 3;46(2):255–68. doi: 10.1038/s41366-021-00993-1 (PMC8794783; doi:10.1038/s41366-021-00993-1)

A)

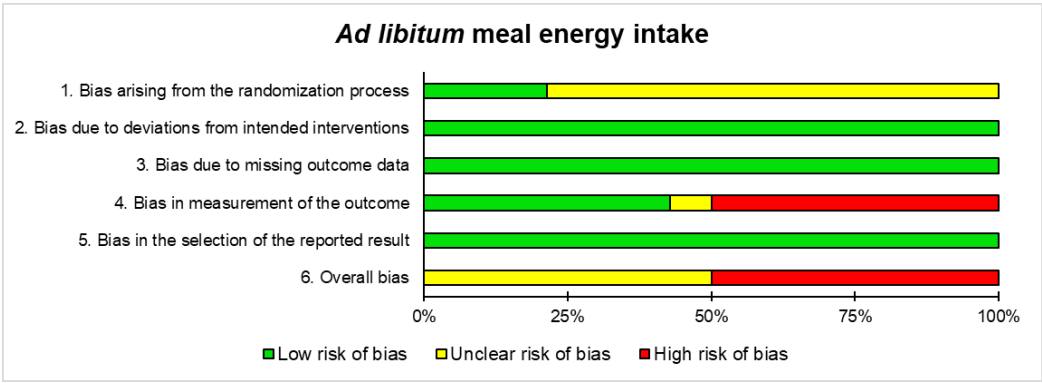

B)

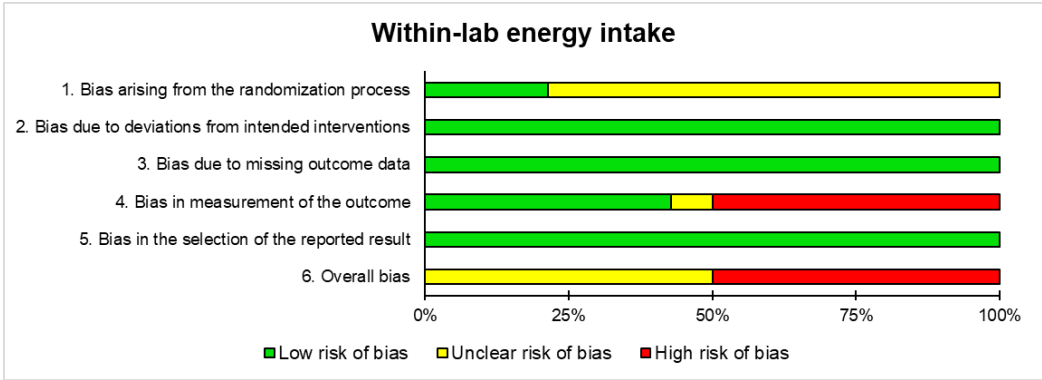

C)

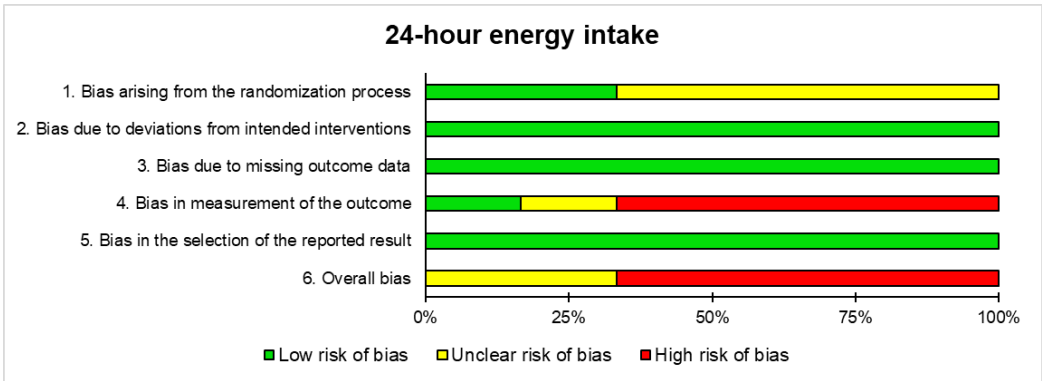

D)

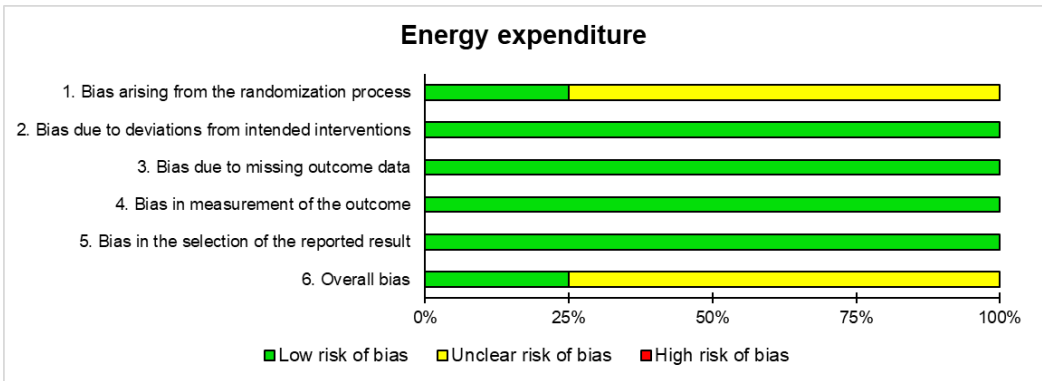

E)

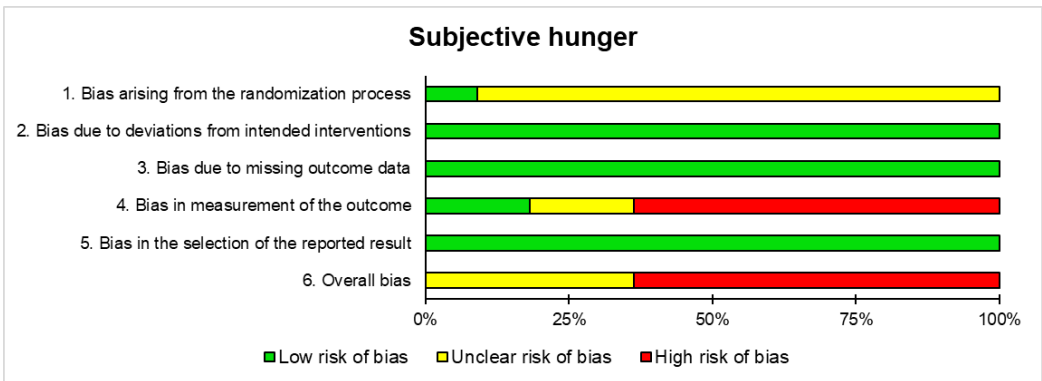

Supplement: Supplementary file 8 — Supplementary Material [file 41366_2021_993_MOESM8_ESM.pdf]

A)

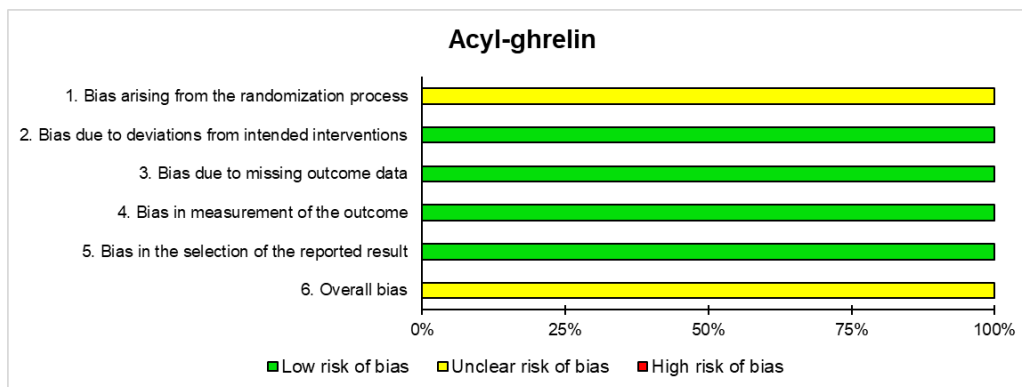

B)

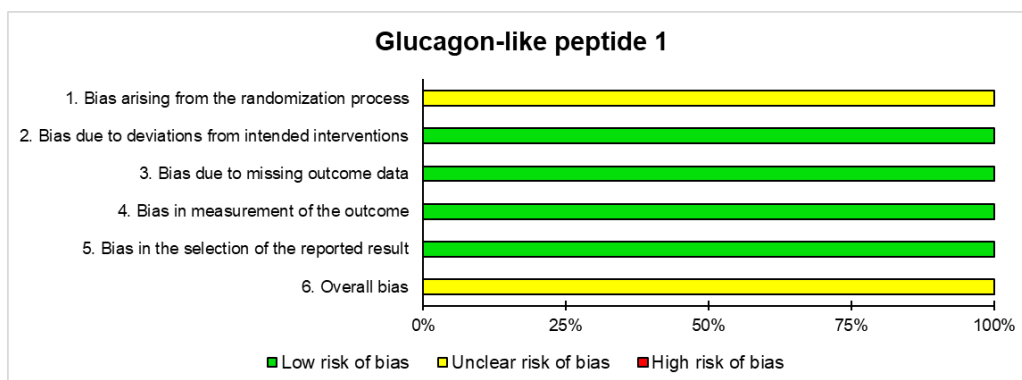

C)

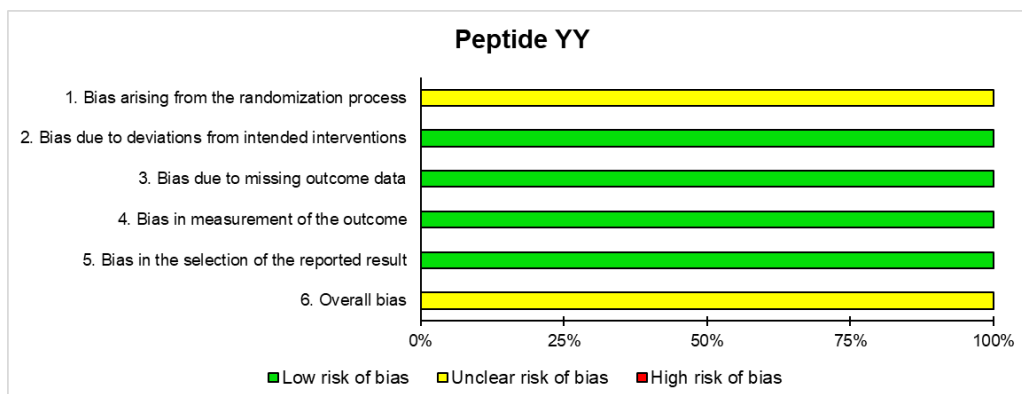

Supplement: Supplementary file 9 — Supplementary Material [file 41366_2021_993_MOESM9_ESM.pdf]

**A)**

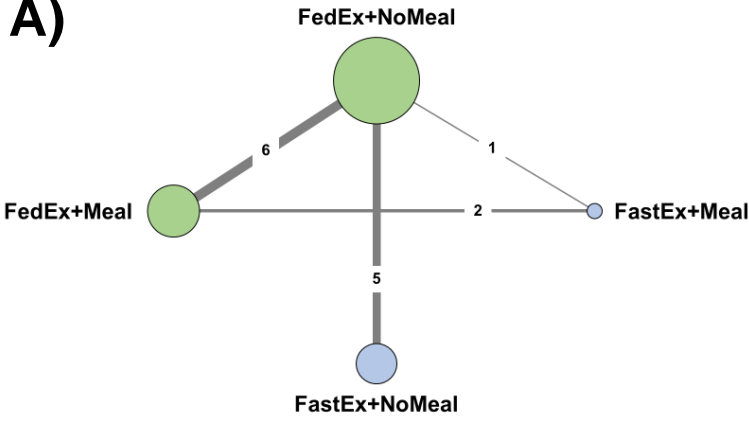

**B)**

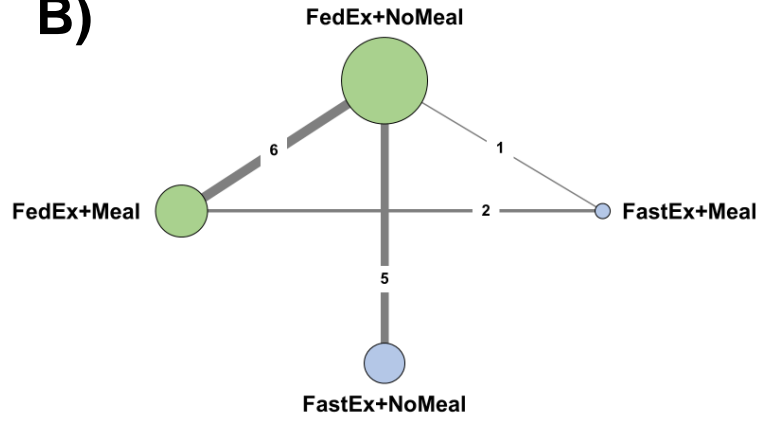

**C)**

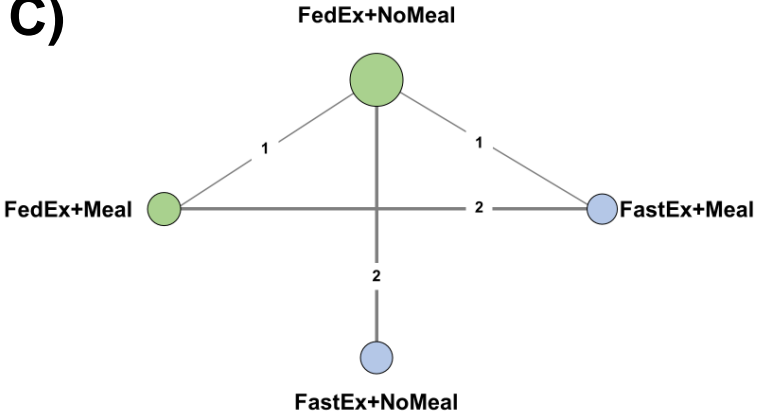

**D)**

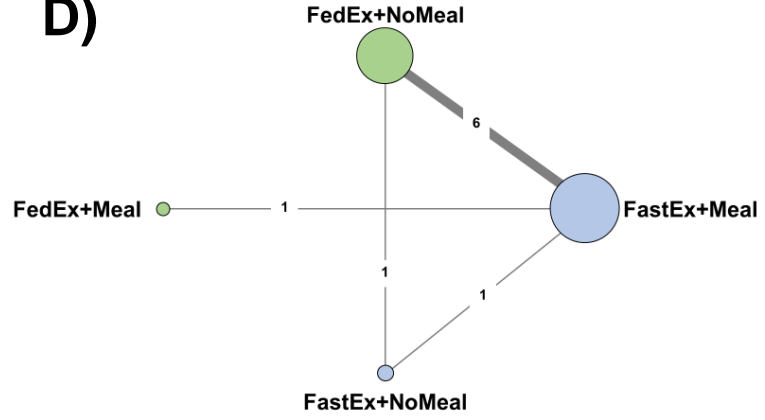

**E)**

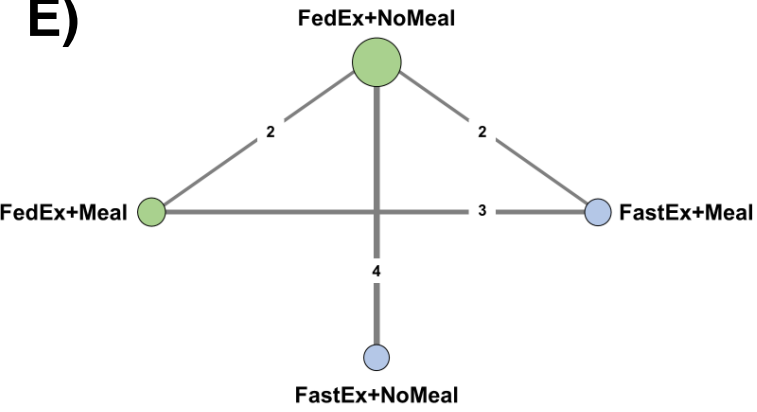

Supplement: Supplementary file 10 — Supplementary Material [file 41366_2021_993_MOESM10_ESM.pdf]

A)

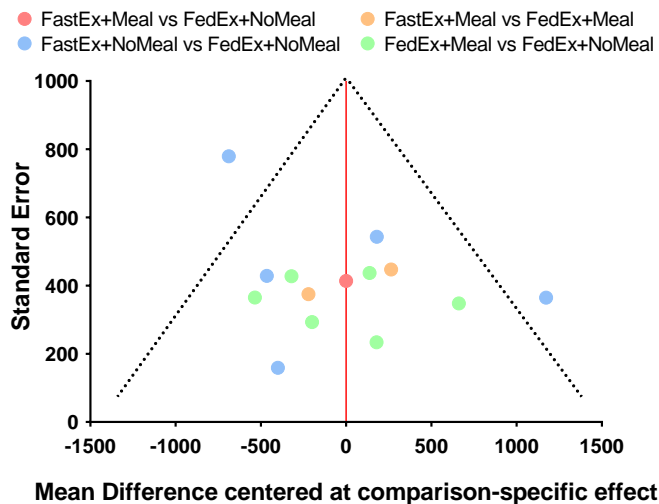

B)

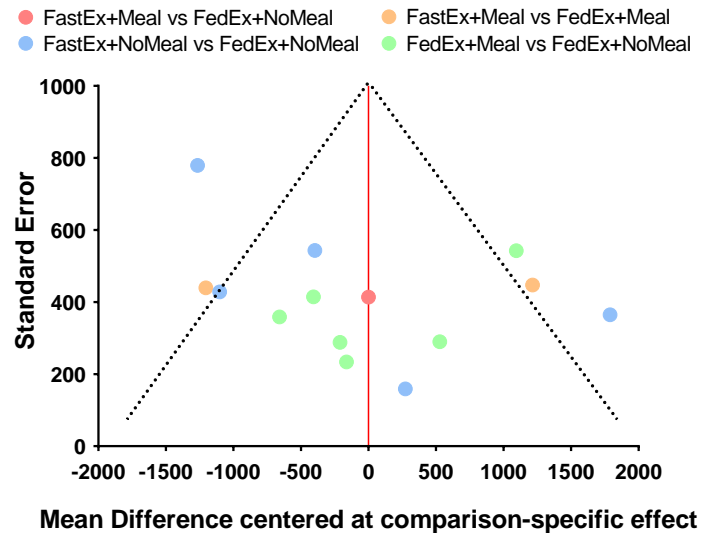

C)

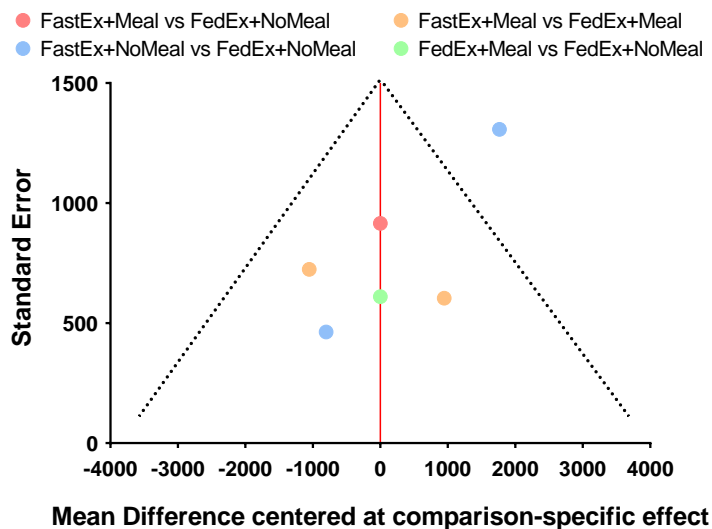

D)

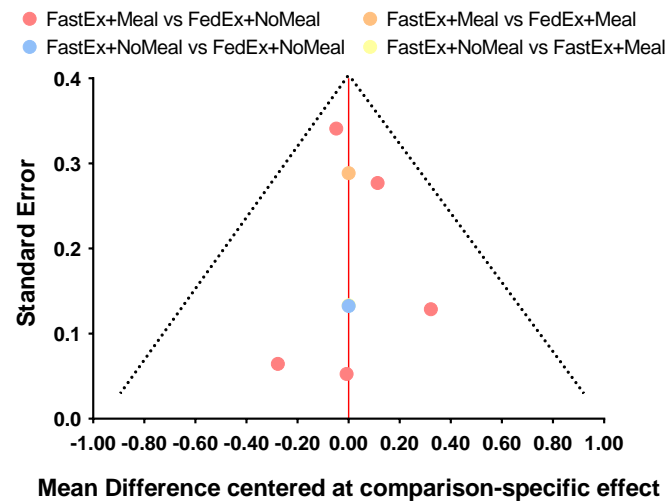

E)

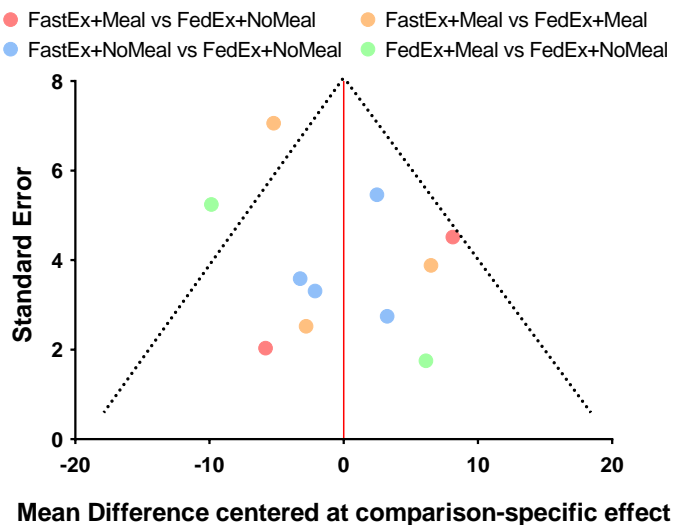

Supplement: Supplementary file 12 — Supplementary Material [file 41366_2021_993_MOESM12_ESM.pdf]

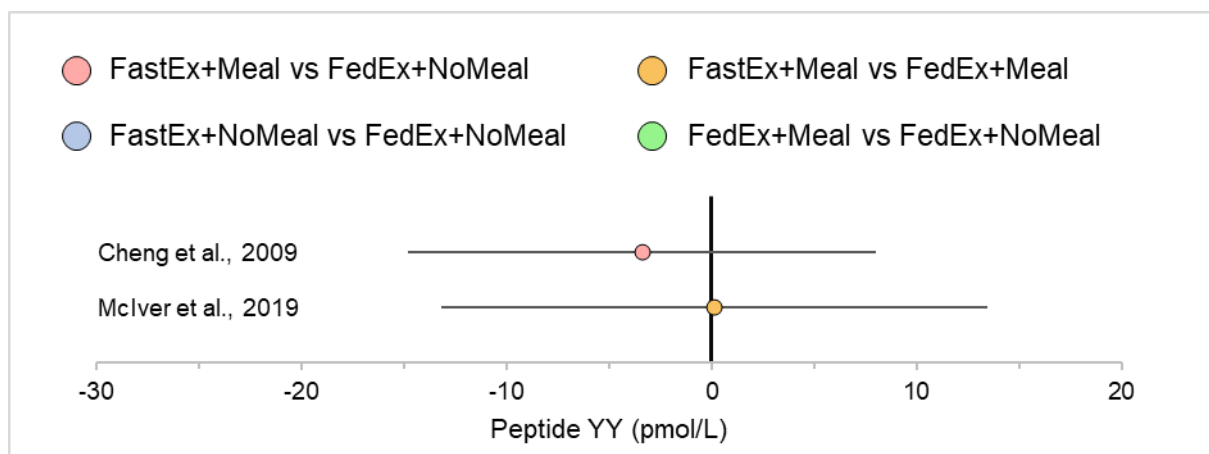

Supplement: Supplementary file 19 — Supplementary Material [file 41366_2021_993_MOESM19_ESM.pdf]
