# Supplementary material for: The acute effect of fasted exercise on energy intake, energy expenditure, subjective hunger and gastrointestinal hormone release compared to fed exercise in healthy individuals: a systematic review and network meta-analysis
Source: Int J Obes (Lond). 2021 Nov 3;46(2):255–68. doi: 10.1038/s41366-021-00993-1 (PMC8794783; doi:10.1038/s41366-021-00993-1)
Supplement: Supplementary file 11 — Supplementary Material [file 41366_2021_993_MOESM11_ESM.pdf]

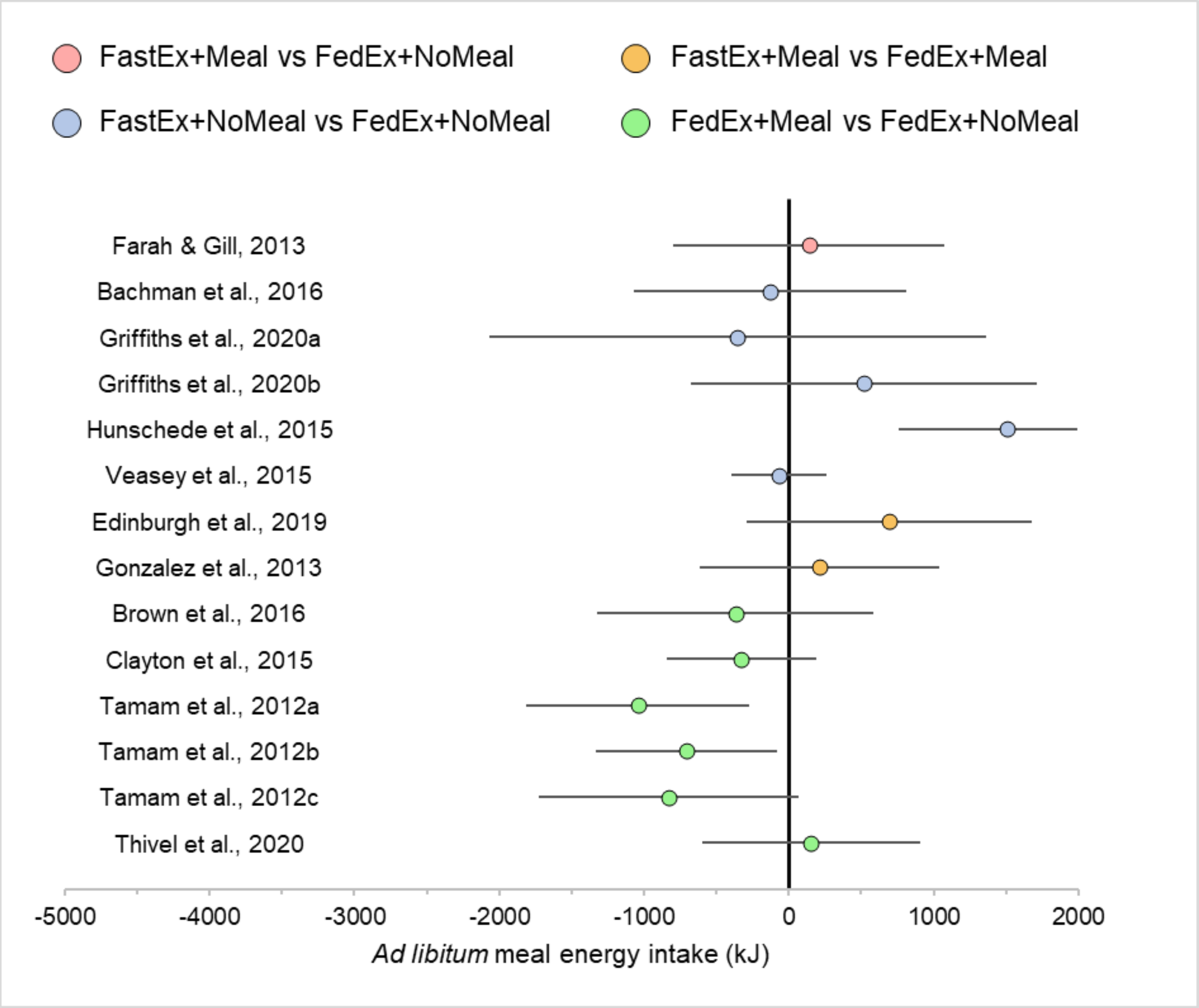
FastEx+Meal vs FedEx+NoMeal
FastEx+Meal vs FedEx+Meal
FastEx+NoMeal vs FedEx+NoMeal
FedEx+Meal vs FedEx+NoMeal
Farah & Gill, 2013
Bachman et al., 2016
Griffiths et al., 2020a
Griffiths et al., 2020b
Hunschede et al., 2015
Veasey et al., 2015
Edinburgh et al., 2019
Gonzalez et al., 2013
Brown et al., 2016
Clayton et al., 2015
Tamam et al., 2012a
Tamam et al., 2012b
Tamam et al., 2012c
Thivel et al., 2020
-5000
-4000
-3000
-2000
-1000
0
1000
2000
Ad libitum meal energy intake (kJ)
