# Supplementary material for: The acute effect of fasted exercise on energy intake, energy expenditure, subjective hunger and gastrointestinal hormone release compared to fed exercise in healthy individuals: a systematic review and network meta-analysis
Source: Int J Obes (Lond). 2021 Nov 3;46(2):255–68. doi: 10.1038/s41366-021-00993-1 (PMC8794783; doi:10.1038/s41366-021-00993-1)
Supplement: Supplementary file 14 — Supplementary Material [file 41366_2021_993_MOESM14_ESM.pdf]

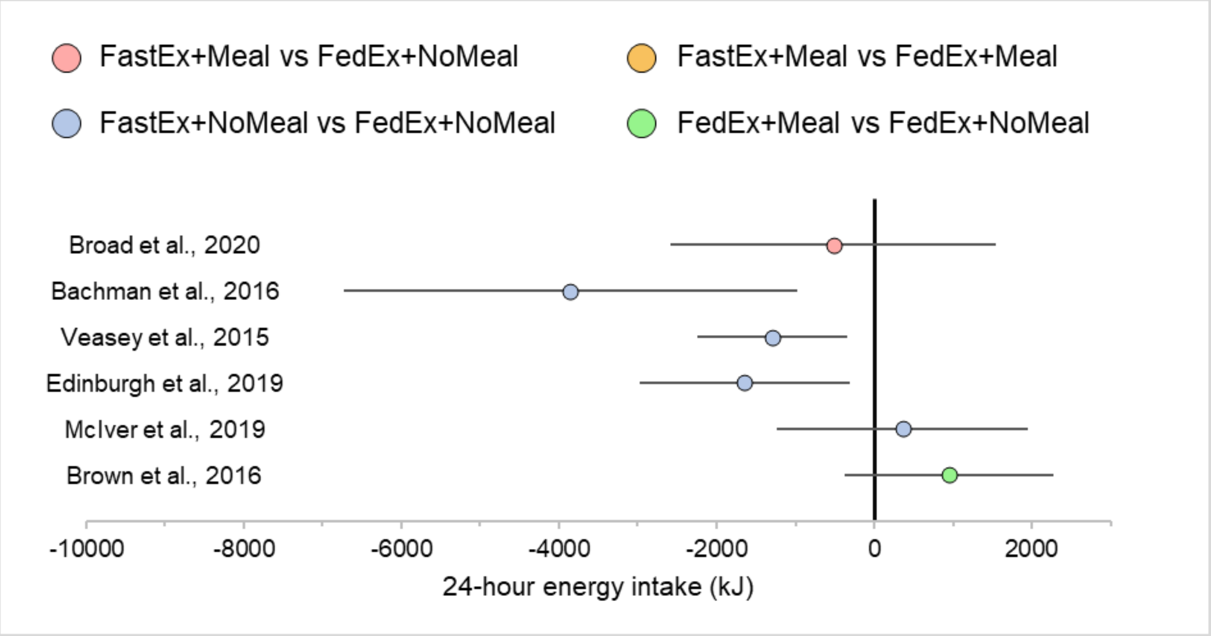
FastEx+Meal vs FedEx+NoMeal
FastEx+Meal vs FedEx+Meal
FastEx+NoMeal vs FedEx+NoMeal
FedEx+Meal vs FedEx+NoMeal
Broad et al., 2020
Bachman et al., 2016
Veasey et al., 2015
Edinburgh et al., 2019
McIver et al., 2019
Brown et al., 2016
-10000
-8000
-6000
-4000
-2000
0
2000
24-hour energy intake (kJ)
