# Supplementary material for: The acute effect of fasted exercise on energy intake, energy expenditure, subjective hunger and gastrointestinal hormone release compared to fed exercise in healthy individuals: a systematic review and network meta-analysis
Source: Int J Obes (Lond). 2021 Nov 3;46(2):255–68. doi: 10.1038/s41366-021-00993-1 (PMC8794783; doi:10.1038/s41366-021-00993-1)
Supplement: Supplementary file 15 — Supplementary Material [file 41366_2021_993_MOESM15_ESM.pdf]

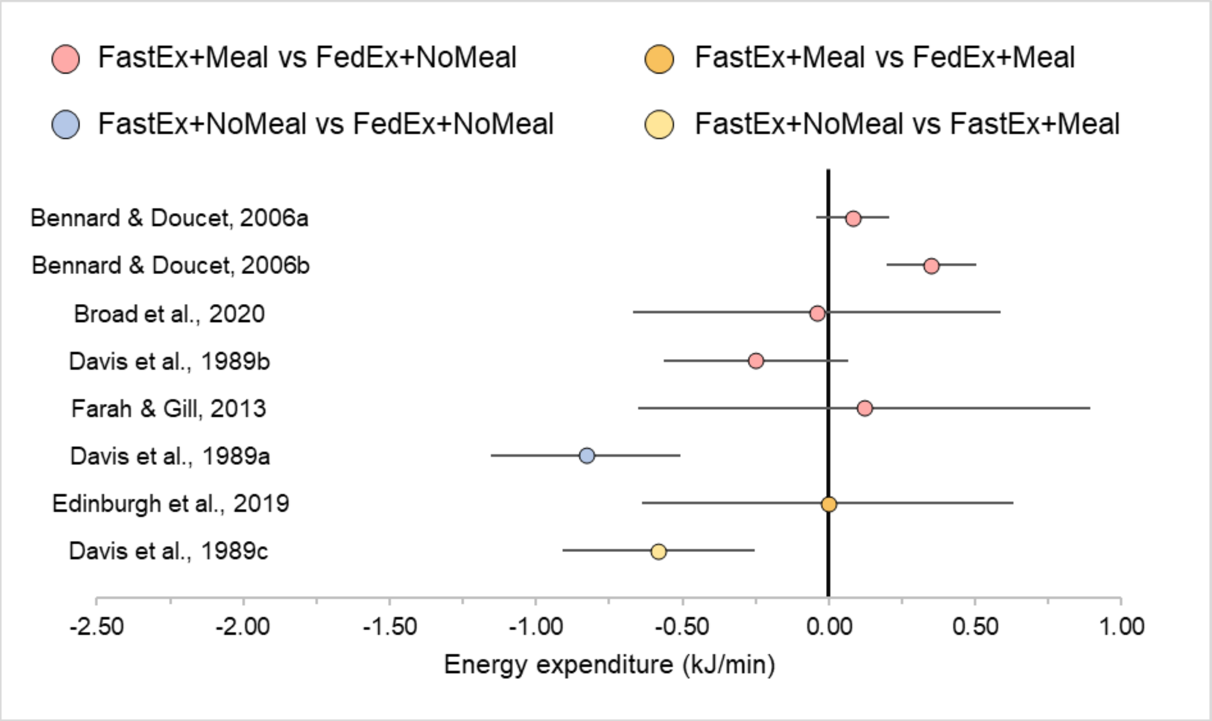

FastEx+Meal vs FedEx+NoMeal
FastEx+Meal vs FedEx+Meal
FastEx+NoMeal vs FedEx+NoMeal
FastEx+NoMeal vs FastEx+Meal
Bennard & Doucet, 2006a
Bennard & Doucet, 2006b
Broad et al., 2020
Davis et al., 1989b
Farah & Gill, 2013
Davis et al., 1989a
Edinburgh et al., 2019
Davis et al., 1989c
-2.50
-2.00
-1.50
-1.00
-0.50
0.00
0.50
1.00
Energy expenditure (kJ/min)
