# Supplementary material for: The acute effect of fasted exercise on energy intake, energy expenditure, subjective hunger and gastrointestinal hormone release compared to fed exercise in healthy individuals: a systematic review and network meta-analysis
Source: Int J Obes (Lond). 2021 Nov 3;46(2):255–68. doi: 10.1038/s41366-021-00993-1 (PMC8794783; doi:10.1038/s41366-021-00993-1)
Supplement: Supplementary file 16 — Supplementary Material [file 41366_2021_993_MOESM16_ESM.pdf]

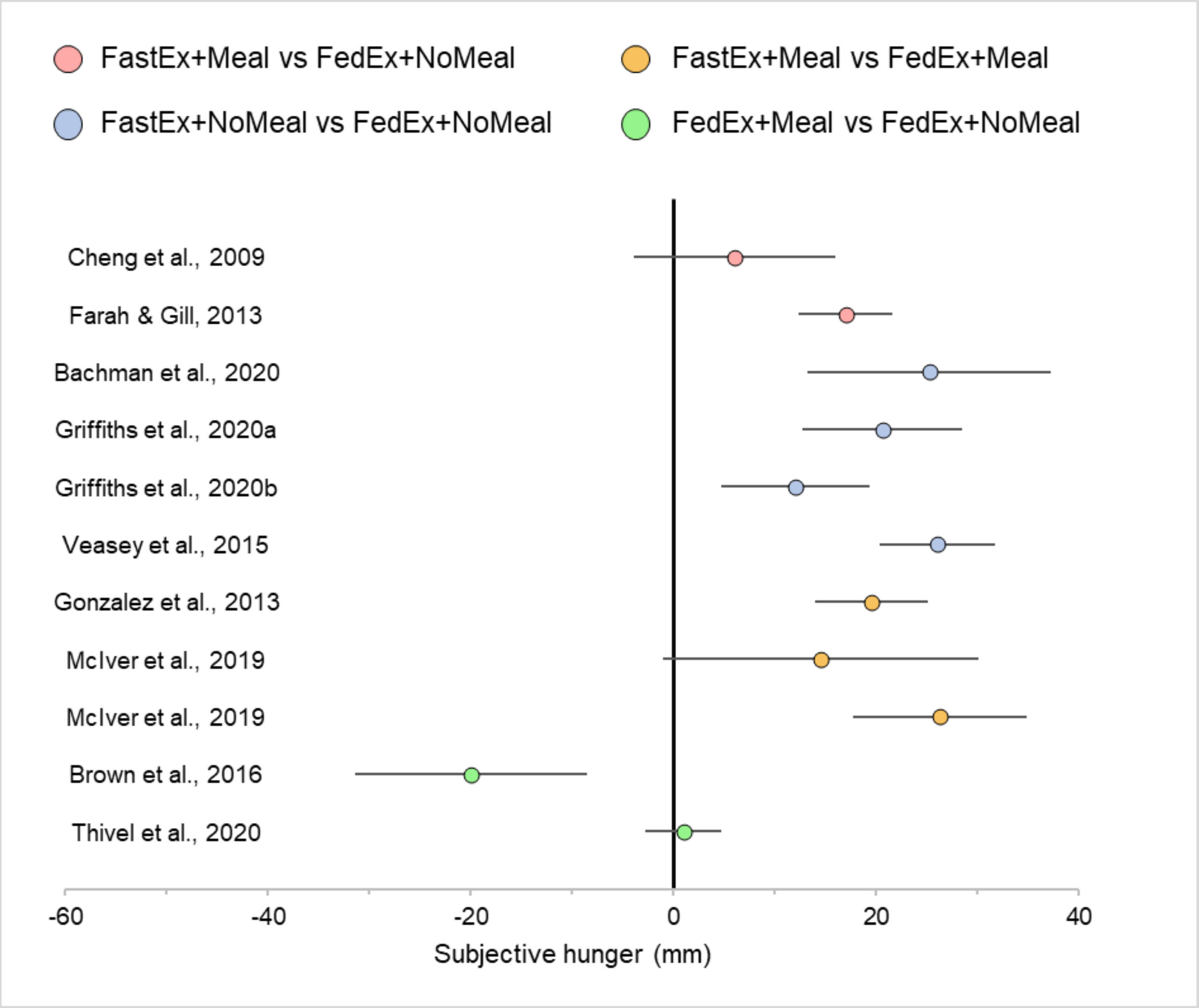
FastEx+Meal vs FedEx+NoMeal
FastEx+Meal vs FedEx+Meal
FastEx+NoMeal vs FedEx+NoMeal
FedEx+Meal vs FedEx+NoMeal
Cheng et al., 2009
Farah & Gill, 2013
Bachman et al., 2020
Griffiths et al., 2020a
Griffiths et al., 2020b
Veasey et al., 2015
Gonzalez et al., 2013
McIver et al., 2019
McIver et al., 2019
Brown et al., 2016
Thivel et al., 2020
-60
-40
-20
0
20
40
Subjective hunger (mm)
