# Supplementary material for: The acute effect of fasted exercise on energy intake, energy expenditure, subjective hunger and gastrointestinal hormone release compared to fed exercise in healthy individuals: a systematic review and network meta-analysis
Source: Int J Obes (Lond). 2021 Nov 3;46(2):255–68. doi: 10.1038/s41366-021-00993-1 (PMC8794783; doi:10.1038/s41366-021-00993-1)
Supplement: Supplementary file 18 — Supplementary Material [file 41366_2021_993_MOESM18_ESM.pdf]

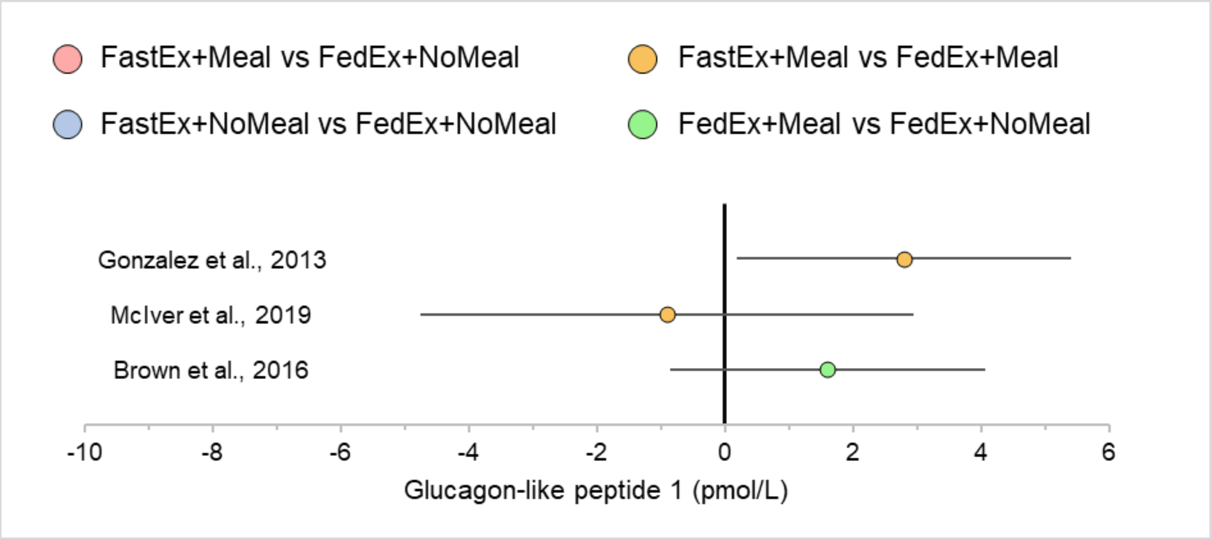
FastEx+Meal vs FedEx+NoMeal
FastEx+Meal vs FedEx+Meal
FastEx+NoMeal vs FedEx+NoMeal
FedEx+Meal vs FedEx+NoMeal
Gonzalez et al., 2013
McIver et al., 2019
Brown et al., 2016
-10
-8
-6
-4
-2
0
2
4
6
Glucagon-like peptide 1 (pmol/L)
